# Motivations to reciprocate cooperation and punish defection are calibrated by estimates of how easily others can switch partners

**DOI:** 10.1371/journal.pone.0267153

**Published:** 2022-04-19

**Authors:** Sakura Arai, John Tooby, Leda Cosmides

**Affiliations:** 1 Center for Evolutionary Psychology, University of California, Santa Barbara, California, United States of America; 2 Department of Psychological & Brain Sciences, University of California, Santa Barbara, California, United States of America; 3 Department of Anthropology, University of California, Santa Barbara, California, United States of America; Zhejiang University of Finance and Economics, CHINA

## Abstract

Evolutionary models of dyadic cooperation demonstrate that selection favors different strategies for reciprocity depending on opportunities to choose alternative partners. We propose that selection has favored mechanisms that estimate the extent to which others can switch partners and calibrate motivations to reciprocate and punish accordingly. These estimates should reflect default assumptions about *relational mobility*: the probability that individuals in one’s social world will have the opportunity to form relationships with new partners. This prior probability can be updated by cues present in the immediate situation one is facing. The resulting estimate of a partner’s outside options should serve as input to motivational systems regulating reciprocity: Higher estimates should down-regulate the use of sanctions to prevent defection by a current partner, and up-regulate efforts to attract better cooperative partners by curating one’s own reputation and monitoring that of others. We tested this hypothesis using a Trust Game with Punishment (TGP), which provides continuous measures of reciprocity, defection, and punishment in response to defection. We measured each participant’s perception of relational mobility in their real-world social ecology and experimentally varied a cue to partner switching. Moreover, the study was conducted in the US (*n* = 519) and Japan (*n* = 520): societies that are high versus low in relational mobility. Across conditions and societies, higher perceptions of relational mobility were associated with increased reciprocity and decreased punishment: i.e., those who thought that others have many opportunities to find new partners reciprocated more and punished less. The situational cue to partner switching was detected, but relational mobility in one’s real social world regulated motivations to reciprocate and punish, even in the experimental setting. The current research provides evidence that motivational systems are designed to estimate varying degrees of partner choice in one’s social ecology and regulate reciprocal behaviors accordingly.

## 1 Introduction

### 1.1 Evolutionary models of dyadic cooperation

The evolution of dyadic cooperation has been explored through evolutionary game theory since the 1970s. A consistent finding is that decision rules that cause cooperation can evolve and be maintained in a population by natural selection if agents can implement a strategy for *conditional cooperation*. These are strategies that direct benefits to agents who cooperate rather than those who defect. Defectors—*cheaters*—are individuals who accept the benefits of cooperation but fail to provide sufficient benefits in return, either by not reciprocating at all or by reciprocating too little [1]. There are, however, many different strategies for conditional cooperation. Which ones are favored by selection depends on the social ecology—especially on the extent to which it provides options for switching partners.

In early models of conditional cooperation—also known as *reciprocity*—agents were not permitted to choose partners or to avoid defectors by switching partners. Agents were randomly paired with their partners, and they interacted with each partner repeatedly. They could recognize and remember (at least some of) their history of interaction with a given partner, and use that information to decide whether to cooperate or defect in a given round. This social ecology favored *sanction-based strategies*, such as TIT FOR TAT, which cooperates when their partner delivers benefits and defects when that partner defects [2]. These strategies are stable against invasion by strategies that defect because they respond to defection by withholding benefits or inflicting costs (“punishment”), and they resume cooperation only after the defecting partner cooperates again. When agents with sanction-based strategies are paired with other conditional cooperators, they repeatedly harvest the benefits of mutual cooperation, allowing them to outcompete strategies that defect. Because switching partners to avoid defectors is not an option in these models, Hammerstein and Noë [3] call them “partner control models without outside options.” In this social ecology, cooperation is maintained by natural selection because agents monitor their partner’s behavior and “control” it through positive and negative sanctions. Empirical work suggests that some non-human organisms use sanction-based strategies in reciprocal cooperation [4–6].

Sanction-based strategies differ in detail: For example, some leave defectors [7–12], some cooperate contingently, withdrawing cooperation after one defection [2], and yet others require several defections, thereby maintaining cooperation with conditional cooperators who defected by mistake [13]. But one’s reputation as a cooperator, defector, or punisher plays no role in these strategies, beyond the history of interaction remembered by one’s current partner.

In the 1990s, evolutionary scientists began to explore selection in *biological markets*: social ecologies in which agents can leave one cooperative partner and choose another [14–16]. These *partner choice models* assume that agents can infer and represent the reputation of multiple potential partners based on available information, such as their behavior when interacting with other individuals (did they cooperate? defect? punish?) or other observable traits (e.g., skill procuring valued resources). They also assume that agents can use reputation information in deciding whether to stay with their current partner or switch to a different one. In these models, competition to be chosen—or retained—as a cooperative partner “controls” defection and stabilizes cooperation by the threat of partner switching [3]. Partners who defect are abandoned for partners who are more likely to provide benefits.

A social ecology in which agents can switch partners favors *reputation-based strategies*: ones that (i) prefer partners who are likely to reciprocate—ones with a reputation as a reliable cooperator, and (ii) manage their reputation to attract valuable cooperative partners [17–19]. Empirical studies have shown that many organisms, including humans, behave as if they have evolved reputation-based strategies [20–22].

### 1.2 Strategies for cooperation under different conditions: Social ecologies with low versus high partner choice

The strategies favored by selection differ in these two contexts because they pose quite different adaptive problems, especially regarding the best response to defection [23–25]. The early models, which prevent partner choice entirely, are the most extreme version of a *low partner choice ecology*. In this social ecology, the only way to minimize the costs of defections is to sanction the defecting partner. If the partner is not reciprocating at all, one can go on strike—refuse to provide benefits until the partner starts to cooperate—or punish the defection by inflicting a cost on the partner, possibly at some cost to oneself. Neither party realizes the benefits of mutual cooperation until the partner responds by cooperating. If the partner is under-reciprocating, one can down-regulate the benefits one provides successively, until the partner responds by providing more in return. But one does not have the option of switching to a more rewarding partner.

Selection pressures are different in a *high partner choice ecology*. The most extreme version is a social ecology in which many alternative cooperative partners are available, information about their reputations is free, and there is no cost to switching partners. Under these conditions, the opportunity cost of staying with a partner who defects or under-reciprocates is high. An *opportunity cost* is the benefit one would gain by choosing the best alternative option; in this case, the opportunity cost is equal to the benefits you would harvest by interacting with the most cooperative alternative partner *who is willing to interact with you*. The opportunity cost is high when the payoff of remaining with a partner who defects or under-reciprocates is lower than the payoff of switching to a more cooperative partner.

High opportunity costs select against sanction-based strategies—even those that never pay a cost to punish a defector. When you down-regulate or withdraw cooperation to reform an uncooperative partner, you are forgoing the benefits of mutual cooperation that you could gain by interacting with a different, more cooperative partner. In a high partner choice ecology, abandoning your current partner for a more cooperative one is more fitness-promoting than retaining and trying to reform an uncooperative partner. This is true even if your current partner does reciprocate; selection favors switching partners when your best outside option provides higher payoffs than your current partner.

### 1.3 The problem of being chosen

Switching to a new, more cooperative partner will not be an option, however, if high value cooperative partners do not want to interact with you. Because valuable cooperative partners will prefer to interact with the most rewarding partners available to them, developing a reputation for cooperation is a way of competing for good partners in ecologies where partner choice is high [26, 27]. But what kind of reputation will attract valuable cooperative partners?

#### 1.3.1 Reputation for providing benefits

The most straightforward way to acquire a reputation as a good cooperator is to resist temptations to cheat and behave cooperatively [24]. Enhancing this reputation can be accomplished by providing as much—or more than others in your social ecology [22]; initiating cooperative relationships by delivering benefits [28]; or demonstrating skill at acquiring resources [29]. People invest in acquiring a cooperative reputation, even in the laboratory: They are more generous in cooperative games when they can be observed by third parties [30]. And partner choice can elicit “competitive altruism”: When the observer will have the opportunity to choose a cooperative partner, people are more generous than when partners are fixed or randomly assigned [22, 31–33].

#### 1.3.2 Reputation for inflicting negative sanctions?

A reputation for sanctioning failures to reciprocate may deter defection whether partner choice is low or high. But does it harm your reputation as a valuable cooperator in high partner choice ecologies?

Not all failures to reciprocate arise from a disposition or intent to profit from the temptation to cheat. An otherwise good cooperator can make a mistake or be temporarily unable to reciprocate due to injury or lack of resources [34]. Under these circumstances, sanctioning a failure to reciprocate can trigger defection in return, jeopardizing the flow of benefits that result from mutual cooperation [35]. Sanctioning mistakes carries additional risks when partner choice is high: An otherwise good cooperator may leave you for a partner who is less punitive and more rewarding. Sometimes there is a downside to sanctioning intentional defections: An occasional defector who provides higher net benefits than any of your outside options may leave for a more forgiving partner.

When partner choice is high, imposing negative sanctions not only risks a current relationship; it could threaten future ones as well. There can be reputational costs to withdrawing benefits and, especially, to inflicting punishment [36]. Few studies directly compare the effects of these two methods of sanctioning in cooperative interactions. But the reputational consequences of punishing have been explored in a handful of studies in which participants witness several potential partners who vary in how punitive they are toward others. When asked if they wanted to interact with a specific partner in various economic games, participants were less likely to choose punitive over non-punitive partners as recipients [37–39], although punishers were sometimes more likely to be preferred as providers [37] (but see [38, 39]). Potential partners who sanctioned by punishing had a worse reputation than those who sanctioned by rewarding [38, 39]. In another study, punishers were trusted less (and proved less trustworthy) than non-punishers, whereas generous behavior elicited trust [40].

Taken together, these studies suggest that inflicting punishment can decrease one’s desirability as a potential cooperative partner. In high partner choice ecologies, this reputational cost may not be compensated by eliciting more cooperation from defectors than withdrawing benefits does, at least when strangers interact. In a repeated prisoners dilemma (PD) in which there were two methods for sanctioning a partner—inflicting punishment or withdrawing for one round—punishment did not elicit more cooperation than withdrawing cooperation [23].

In sum, what counts as adaptive behavior varies with social ecology. When partner choice is limited, the only way to elicit cooperation from an uncooperative partner is to withhold benefits or inflict punishment. But these negative sanctions may be unnecessary—and possibility counter-productive—in high partner choice ecologies, where one’s bargaining power depends on having good outside options: alternative partners who are not only cooperative, but also willing to choose you. These considerations raise an under-explored question: Does information about partner choice in one’s local ecology calibrate motivations to cooperate and punish?

### 1.4 Estimating degrees of partner choice

Computational systems that generate motivations to reciprocate, defect, or punish regulate cooperative behavior. Their evolved design should reflect selection pressures common in the social ecologies of our group-living hominin ancestors. Did these social ecologies select for designs that implement sanction-based strategies or reputation-based strategies?

The evolutionary models discussed above represent two extremes on a partner choice continuum. At one extreme are models in which one can either engage in a relationship with a single partner or forgo cooperation entirely. At the other extreme are models in which many cooperation partners are available and switching partners is cost-free. But neither extreme was common during hominin evolution.

Hunter-gatherers are rarely forced to engage with one and only one cooperative partner, even when they live in very small bands. They usually have the option to forage individually rather than cooperatively, or to cooperate exclusively with kin (which does not require reciprocation to be advantageous) [41–43]. Nor did they have access to an unlimited number of partners with zero cost of switching. Most social ecologies were intermediate between these two extremes.

Does this imply that human motivations to reciprocate, defect, or punish are tuned to a social ecology with a single, intermediate level of partner choice? Not necessarily. The availability of cooperative partners, i.e., the pool of potential partners for dyadic cooperation, depended, in part, on band size, which varied with foraging conditions from ~25 men, women, and children—2 to 3 extended families—to as many as 500 for more sedentary hunter-gatherers and for nomadic bands when they periodically aggregate [44]. This variation could occur within a lifetime (with changes in season, rainfall, and game dispersal) and over generations: From the first appearance of anatomically modern humans, global climate has alternated between ice ages and warming periods; sometimes changed by 10°C (18°F) within a few decades [45]; and varied with latitude as hominins dispersed across the globe. We propose that this variation selected for motivational systems that treat partner choice as a continuous variable and adjust behavior accordingly. All else equal, the perception that other people can easily switch partners should up-regulate motivations to reciprocate their help and down-regulate motivations to sanction their defections.

This calibration requires mechanisms that can estimate the degree of partner choice in the situation one is facing. This can be decomposed into two questions: (i) How much partner choice is there in my local social ecology *in general*, and (ii) what are the prospects for partner switching *right now*, in my immediate situation? Estimating the probability that one’s current partner can switch to a better outside option is a judgment made under uncertainty. A mechanism that is well-designed for estimating this probability might implement a Bayesian updating process [35, 46].

When you have no previous history with a new person, and no specific knowledge about that person’s value as a cooperator, the prior probability that this person will be able to switch to a better outside option should be based on estimates of partner choice in your local social ecology. This estimate reflects the prospects for switching partners for a person randomly drawn from that ecology. It can be based on a variety of cues, such as how many individuals you encounter on a regular basis, how frequently you encounter new people, how easy it is to change social groups, how trustworthy the average person is, the prevalence of exploitive behavior (including violence), whether the environment is resource-rich or resource-poor, and the number of individuals who can afford to share resources with others.

This prior can be updated based on cues present in the immediate situation. These cues might speak to qualities of the *person* or features of the *situation*. Qualities of the person relevant to their outside options are judged from thin information: In ultimatum games, participants who see photos of their partners’ faces offer more to those whose faces had been rated (by others) as more attractive, kind, cooperative, healthy, trustworthy, higher in status, and (surprisingly) more productive as a cooperative forager [29, 47]. A prior based on social ecology could be updated based on features of the situation as well: Are we temporarily isolated or are alternative partners available right now [48]? Does this situation draw people from my ingroup or an outgroup [49]? A mechanism that is well-designed for estimating the probability that a specific partner will switch should use person-specific and situation-specific cues to adjust a prior based on social ecology upward or downward. The resulting estimate—a posterior probability—represents your current partner’s ability to leave you for a better partner, compared to an average individual from your local ecology.

This posterior probability should reflect both the local social ecology and cues about the immediate situation. When cues in the immediate situation are minimal, the posterior probability should be closer to the prior probability, which was based on the local social ecology. As you gain more experience of a particular partner, the posterior probability may depart more from that prior. In either case, the posterior probability that your current partner can easily switch partners should calibrate your motivations to cooperate with that person and invest in your reputation as a valuable cooperator.

### 1.5 Relational mobility reflects partner choice in a social ecology

For a given social ecology, what is the prior probability that a newly encountered individual will have the opportunity to leave a cooperative partnership with you to form a new one? This probability is proportional to *relational mobility*: the number of opportunities in a given society for individuals to form new relationships [50]. The more such opportunities the average person has, the greater the degree of partner choice in that society.

A twelve-item scale created by Yuki, Schug, and colleagues [50] measures people’s perceptions of relational mobility. It first prompts the rater to think of others in their immediate society, such as people in their workplace or neighborhood. It then asks how much the rater agrees with statements about other people, such as “They have many chances to get to know other people,” “There are few opportunities for these people to form new friendships” (reverse-scored), “If they did not like their current groups, they would leave for better ones.” The relational mobility scale measures the extent to which other people are seen as having many alternative partners to choose from in the context specified (e.g., group members, friends, or other relationships).

Perceptions of relational mobility vary across societies: Average scores are higher in the US than in Japan, for example [51]. These scores predict societal differences in motivations that are theoretically relevant to partner choice. In their review of the literature on relational mobility, Yuki, Schug, and colleagues summarized how people in different societies react to incentives created by levels of relational mobility [52, 53]. In societies where people believe relational mobility is high, they are geared toward (i) looking for new partners and evaluating their qualities, as well as (ii) advertising one’s qualities as a partner and displaying commitment to desirable partners [51, 54, 55]. These behavioral tendencies suggest the operation of reputation-based strategies: efforts to choose better partners based on their reputation and to be chosen by improving one’s reputation as a valuable cooperator.

Where relational mobility is low, people behave as if they have few outside options. Oishi et al. [53] report that people in these social ecologies are more likely to (i) invest in maintaining cooperation within small, close-knit groups, and (ii) avoid being excluded from these close-knit cooperative relationships by cooperating rather than defecting with their current partners. They behave as if their partners are enacting sanction-based strategies: They cooperate with existing partners by default, and assume their partners are ready to respond to defection by imposing negative sanctions [49, 56].

The prior probability that other people in your social ecology can switch partners varies with relational mobility: the number of opportunities the average person has to form new relationships. *Perceptions* of relational mobility are mental representations of this prior probability: They reflect the mind’s estimate of how much partner choice others can exercise in your local social ecology. If the mind is designed to treat partner choice as a continuous variable, then measures of relational mobility should regulate motivations to reciprocate, defect, and punish, especially when interacting with people you do not know.

### 1.6 The current experiment

Here we investigate the design of motivational systems that regulate dyadic cooperation. The goal is to see if motivations to cooperate with, punish, and/or switch partners are calibrated by estimates of the degree to which others can exercise partner choice. If the mind is designed to treat partner choice as a continuous variable, these motivations should vary with relational mobility—an estimate of partner choice in one’s local social ecology—and with verbal cues, delivered with the instructions, about partner choice in the immediate situation. As estimates of the probability that a partner can switch increase, we expect concern with one’s reputation as a cooperative partner to increase, leading to more reciprocation and less punishment.

**Fig 1:**
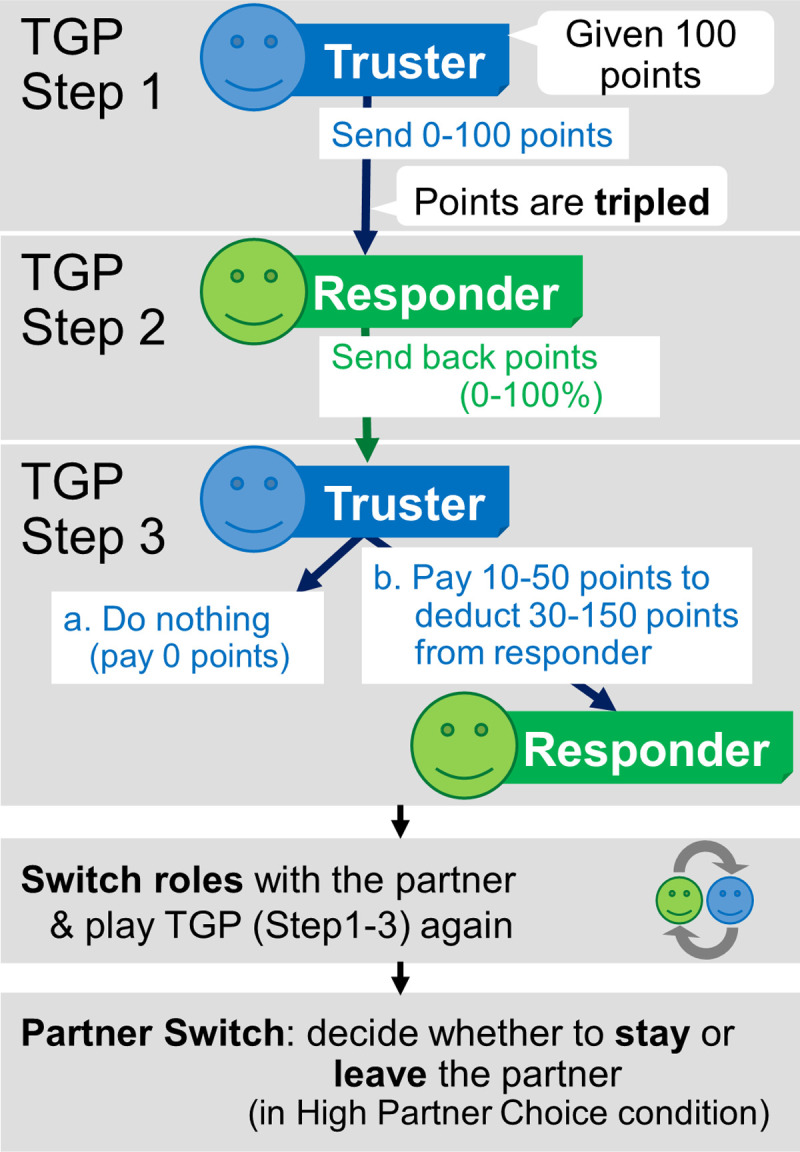
The flow of TGP and partner switching. Participants interacted with their partner in the Trust Game with Punishment (TGP). After interacting once (as the truster or the responder), the participant and the partner switched roles and interacted in the TGP again (order counterbalanced). After interacting with the same partner twice, once in each role, participants in the High Partner Choice condition decided whether they wanted to continue interacting with their current partner in the next TGP, or switch to a new partner. Participants in the Low Partner Choice condition were reminded that they would continue interacting with the same partner.

We proceeded as follows. To measure motivations to cooperate with, punish, and switch partners, we used a game from behavioral economics in which two individuals can benefit by mutual cooperation: a Trust Game with Punishment (TGP) [57]. It provides two interacting individuals—a truster and a responder—an opportunity to benefit each other by reciprocally cooperating (see Fig 1). The truster, who starts with 100 points, decides how many to invest in their partnership. Because the invested points are tripled, both partners can be better off, but only if the responder shares enough of them with the truster (more than 1/3). If the responder gives too little, the truster has an opportunity to punish this decision (see Section 2.2.1).

Each participant completed the relational mobility scale, to measure their estimate of how much partner choice others in general can exercise in their local social ecology. Before the TGP, half were told they could switch partners after 2 rounds of the game (*High Partner Choice* condition), and half were told they would interact with the same partner for the entire study (*Low Partner Choice* condition). These instructions served as cues to partner choice in the immediate situation. After two rounds, participants in the former condition were asked whether they wanted to switch partners.

In addition to measuring individual perceptions of relational mobility, the study was conducted in two countries where perceptions of relational mobility differ on average: the US (high) and Japan (low).

## 2 Materials and methods

### 2.1 Participants

Participants (N = 1039) were from the US (*n* = 519, 53.5% male, *M*_*age*_ = 38.84, *SD*_*age*_ = 11.52), recruited via Amazon Mechanical Turk, and Japan (*n* = 520, 53.8% male, *M*_*age*_ = 41.73, *SD*_*age*_ = 9.64), recruited via an equivalent crowdsourcing website, Lancers, with instructions translated to Japanese by a native speaker (the first author). They were compensated approximately 3 dollars (either in US dollars or Japanese Yen) for their participation in the study, which lasted about 25 minutes.

Those who wished to participate in the study first completed an informed consent form. After the study, participants received a written debriefing about the study design and purposes. They were then asked for consent to use their data; it was explained that they would be compensated regardless of their answer. Fourteen participants who did not provide consent were excluded from the analysis. This study was approved by the Institutional Review Board at University of California, Santa Barbara (Human Subjects Committee). See S1 Appendix for materials and S2 Appendix for data.

### 2.2 Design

There were two experimental conditions: High versus Low Partner Choice. Participants were randomly assigned to one of them. After reading instructions for a TGP [57], they were told that they would be paired with a partner.

In the Low Partner Choice condition, participants were told that they would be interacting with the same partner for the rest of the study. In the High Partner Choice condition, the instructions explained that, after interacting with the same partner twice in two TGPs, they had a choice: (i) They could switch to a new, unknown partner, or (ii) they could remain with their current partner for the next TGP—*but only if that partner chose to remain with them*. Thus, they knew before their first TGP that keeping their current partner might depend on their reputation in that partner’s eyes.

Note that participants in the High Partner Choice condition could decide they wanted to interact with a new partner after the second round, but they were not permitted to choose among alternative partners (and had no information about such partners). This was for strict experimental control, to ensure that the High and Low conditions differed in only one respect: whether people could leave their current partner or not.

#### 2.2.1 Reciprocation and punishment in the TGP

Before the TGP, participants were told that they were going to be given points that could be used during the interaction. They were asked to imagine that the points they earned would be converted to real money at the end of the study. In the TGP, participants experienced both roles, truster and responder (order counterbalanced across participants). The TGP was same as the standard Trust Game, except a punishment phase was added after the responder’s decision [57]. We will use terms such as “reciprocation” and “punishment” to describe the logic of the game, but these terms were not used in the instructions to participants.

Participants were told they would interact with another participant. In reality, they interacted with sham partners simulated by a program. This procedure, which was the only deception in the study, was necessary to examine hypotheses about how people react to different reciprocal behaviors.

Before the interaction began, each participant (real and sham) was given 50 points as “a bonus.” This was done to ensure that trusters had enough points to punish the responder, regardless of how many points the responder returned to the truster.

The TGP had the following structure. The truster was given an endowment of 100 points to send or keep. The truster could send any number of points to the responder, from 0 ≤ P ≤ 100, in 10-point increments. The P points sent to the responder were tripled, and the responder decided what percentage of (now) 3P points to return to the truster. (Options were displayed as both percentages of 3P and points; see S1 Appendix.) The percentage returned is the dependent variable that measures reciprocation by the participant (Dependent Variable [DV] 1: *Reciprocation by the participant*; 0–100% in 10% increments).

If the truster sends nothing to the responder, the truster keeps all 100 points. Sending points is a risky *investment*—because they are tripled, both parties can be better off, but only if the responder sends enough points back to the truster. Any points sent to the responder are at risk because the responder could decide to send nothing back to the truster, or so few points that the truster is worse off than if she had *not* risked the P points that she invested. The truster, whose payoff is [(100 –P) + (.X×3P)], breaks even when the responder returns 1/3 of 3P points (payoff = 100, i.e., 100 –P + P). The truster realizes a positive payoff when the responder returns more than 1/3 of the tripled points and incurs a loss when less than 1/3 of 3P is returned.

After seeing what percentage the responder gave to the truster, the truster had the option to pay 10 points to subtract 30 points from the responder; the truster could pay up to 50 points, in 10-point increments, to subtract up to 150 points from the responder. Note that the instructions referred only to subtracting points; this was not labelled “punishment”. The instructions included examples to make sure that participants understood the consequences of various decisions (see S1 Appendix for the full text of instructions).

When the participant was the responder, the (sham) truster always sent 70 points to the participant (70% of the endowment). These were tripled to 210 points. The participant responded by deciding what percent of these points to return to the truster. If the participant returned less than 50% of the 210 points, there was a 50% chance that the truster would pay 20 points to deduct 60 points from the participant. Participants who returned 50% or more of the points they received were never punished. (Punishing cooperators—*anti-social punishment*—is a rare response in real life for these populations [58, 59]. Our interest herein is motivations to cooperate with or leave partners who punish acts that could be perceived as failures to reciprocate sufficiently.) When the participant was the truster, the responder returned either 50% or 20% of the 3P points that the participant had made available. The participant then decided whether to deduct points from the responder. The number of points the participant paid to deduct points from the responder is the dependent variable that measures the participant’s willingness to punish the partner (DV2: *Amount paid to punish the responder*; 0–50 points).

After the instructions for the TGP, participants had two practice rounds, once as the truster and once as the responder. They then answered five comprehension check questions about the TGP and their experimental condition (see S1 Appendix). About 2% of the initial participants (20 people) failed this check; these individuals did not progress to the TGP phase of the study.

#### 2.2.2 Partner switching after the TGP

After interacting with their partners in the TGP twice—once as a truster and once as a responder—participants were reminded that they were going to play the TGP again. Participants in the Low Partner Choice condition were reminded that they would continue interacting with the same partner. Participants in the High Partner Choice condition were asked whether they would like to stay with their current partner or switch to a different partner (DV3: the decision to switch partners). Before deciding, they were reminded that they would keep the same partner only if both they and their partner chose not to switch. (*N*.*B*.: participants did not have to pay a cost to switch or to stay.) At the point when a third TGP was about to commence, all participants were told that the program had decided that there would be no further rounds of the TGP.

#### 2.2.3 Measures

After the TGP, participants completed the relational mobility (RM) scale twice, in different forms: the original and a modified version (order counterbalanced). The original RM scale asked participants how many opportunities they think people around them have to find new partners (*RM others*): e.g., “It is easy for them to meet new people.” The modified one asked the same questions, but about themselves (*RM self*). The *RM self* scale had the same 12 items as the original scale, except that words referring to others were replaced with words referring to oneself: e.g., “It is easy for me to meet new people." *RM self* was added to control for individual differences in perceptions of one’s own opportunities to find new partners, which need not correspond to estimates of the relational mobility of other people in one’s social ecology. We also recorded which society participants were from (US or Japan).

## 3 Results

Data were analyzed using R 4.0.3 [60]. We examined the effects of the experimental manipulation (High versus Low Partner Choice condition), participants’ relational mobility scores (others and self), and society (US versus Japan) on the three DVs: (i) *Reciprocation by the participant* (DV1), (ii) *Amount paid to punish the responder* (DV2), and (iii) the decision to switch partners (DV3; only in the condition that permitted switching: High Partner Choice).

### 3.1 What predicts the decision to switch partners?

The logic of reputation-based strategies assumes that behavior in reciprocal interactions influences the probability that one’s partner will continue the cooperative relationship or switch to a different partner. So, we first examine whether decisions to reciprocate and punish affected DV3: the participant’s decision to switch partners. The opportunity to switch partners was available only in the High Partner Choice condition (*n* = 505).

Decisions to switch were made after the participant interacted with the same partner twice and experienced both roles: one interaction as truster, the other as responder. When given the option to stay or switch, 37.8% of participants chose to switch partners.

To determine which behaviors influence the decision to switch, we conducted logistic regressions, using the glm function in R [60]. In preliminary analyses, we found that the order of roles—whether the participant played truster or responder first—did not predict decisions to switch partners, nor did the participants’ relational mobility scores (others and self); *p*s > .05. These variables did not improve the Akaike Information Criterion (AIC) either, so they were removed from the model. The model focused on the choices participants made, the responses they experienced, and their society (US = 1, Japan = 0). We checked for multicollinearity using the Variance Inflation Factor (VIF values < 1.6). Although including interactions improved model fit, many of them showed strong multicollinearity even after centering variables by subtracting the mean [61] (VIF > 10), making them difficult to interpret. For this reason, the model below does not include interaction terms.

Five predictor variables from the TGP were entered into the analysis. Two arise from the TGP in which the participant was the responder (and the [sham] truster sent P = 70 points):

(i) *Reciprocation by the participant*: What percent of 3P did the participant return to the truster? (0–100%).
(ii) *Punishment received*: Did the truster punish the participant’s response? (1 = punished, 0 = not punished).

Three predictors arise from the TGP in which the participant was the truster:

(iii) *Trust*: How many points did the participant send to their partner? (P = 0–100 points).
(iv) *Defection by the responder*: Did the (sham) partner respond by reciprocating (returning 50% of 3P) or defecting (returning 20% of 3P)? (50% = 0, 20% = 1).
(v) *Amount paid to punish the responder*: How much did participants pay to punish their partner’s response? (0–50 points).

**Fig 2:**
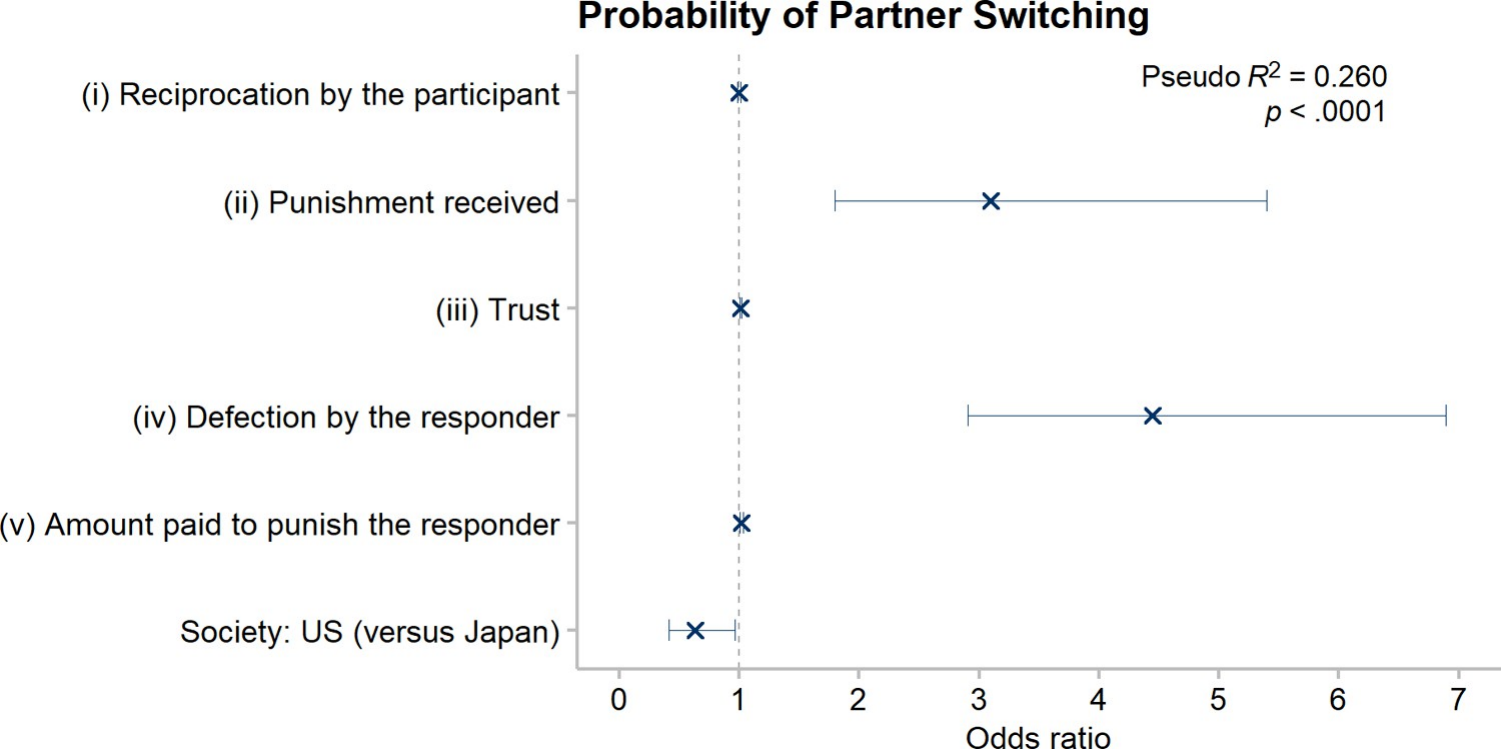
Adjusted odds ratio of each predictor for the decision to switch partners. Estimates of how much each predictor affected the decision to switch partners, when controlling for the five others. An odds ratio greater than 1 indicates a greater likelihood of partner switching; an odds ratio less than 1 indicates a lower probability of partner switching. Bars are 95% confidence intervals. *Reciprocation by the participant* = percent of 3P that the participant returned to the truster (0–100%). *Punishment by the partner* = whether the truster punished the participant’s response (1, 0). *Trust* = P, the number of points the participant sent to the responder (0–100). *Defection by the responder* = the responder defected or reciprocated on the participant (1, 0). *Amount paid to punish the responder* = number of points the participant paid to punish the responder (0–50).

Fig 2 summarizes how each predictor affected the probability (adjusted odds ratio) that the participant would decide to switch partners when controlling for all the others. An odds ratio of 1 means the predictor variable had no independent effect on partner switching. See S3 Appendix for the full model.

#### 3.1.1 Are participants who were punished more likely to switch partners?

When participants were responders, the sham truster could punish them. Fig 2 shows the effect of each predictor variable on the decision to switch partners, when controlling for all the others. It shows that participants who were punished were three times more likely to switch partners than those who were not, controlling for the other predictors: Odds Ratio (OR) = 3.10 (95% CI = [1.80, 5.40]).

Because that odds ratio is based on all participants, it includes those who favored their partner over themselves by returning 50% or more (see Table 1). Their decision to switch partners cannot be a response to being punished, however, because no one who returned 50% or more was ever punished. To see the effect of being punished on partner switching more clearly, the following analyses focus on participants who returned 40% or less, about half of whom were punished.

**Table 1:** Payoffs as a function of percent returned by the responder.

% returned	0%	10%	20%	30%	40%	50%	60%	70%	80%	90%	100%
Points returned to truster	0	21	42	63	84	105	126	147	168	189	210
Truster’s payoff*	30	51	72	93	114	135	156	177	198	219	240
Responder’s payoff	210	189	168	147	126	105	84	63	42	21	0

*The truster can earn 100 by not investing in the responder. Returning at least 40% gives the truster a positive payoff; 40% minimizes the difference in payoffs but favors the responder; 50% or more favors the truster over the responder.

Table 1 shows the payoffs to self (responder) and partner (sham truster) for each choice the responder could make. The sham truster always kept 30 points from the 100-point endowment; the 70 points the truster sent to the responder were tripled to 210 points, and the participant’s task was to decide how many of these points to return to the truster. The responder’s options were limited to 10% increments of 210. What counts as a failure to reciprocate, perhaps worthy of punishment?

Consider these payoffs in light of two concepts of reciprocity discussed in the literature: (i) ensuring the partner gains from having cooperated and (ii) ensuring equal payoffs for both partners [1, 24]. The sham truster could have earned 100 points (the endowment) by investing *nothing* in the responder; by risking 70 points, the sham truster enabled a positive payoff for both parties: the responder and self. A responder who returns more than 70 points—at least 40% of the tripled points—ensures a gain for the truster. Instead of 100 points, the truster will earn from 114 points (40% returned) to 240 points (100% returned).

Returning less than 70 points is a clear-cut case of defection. It creates a loss for the truster, who could have kept all 100 points, leaving the responder with nothing. Having risked 70 points, the truster takes a loss whenever the responder returns 30% (63 points) or less. The more the responder keeps, the worse off the sham truster is for having risked 70 of 100 points. This analysis is general to any positive number of points the truster sends. When the responder returns 40% (or more), the truster’s payoff is positive: (100-P) + .4×3P = 100 + .2P (it would be positive for any return > [1/3]P). The truster’s payoff is negative when the responder returns 30% or less: (100-P) + .3×3P = 100 - .1P.) Twenty-nine percent of responders returned 30% or less (148/505).

When participants were asked “How many points do you want to send back to your partner?”, they chose from a display like the first two rows of Table 1 (shaded in blue). It showed how many points the truster would receive when the participant returned X% of 210 points (see S1 Appendix). Because participants know that the truster risked 70 points, they know the truster realizes a net gain when more than 70 points are returned and a net loss otherwise. They also know the total payoff to the truster is the number of points the participant returns plus the 30 points that the truster kept.

No option results in equal payoffs for both of them. Equal payoffs would require returning 90 points to the sham truster—42.8% of 210—resulting in 120 for each (30 + 90 for truster, 210–90 for responder). Because responders were only allowed to return points in 10% increments, every option favors self over truster (40% or less) or truster over self (50% or more). A participant who views equality as appropriate reciprocation would return 40%: This option ensures a positive payoff for the partner while minimizing the difference in payoffs between self and truster (see below). The majority of responders (259/505 = 51%) chose options that bracketed 42.8% (strict equality) by returning 50% (180/505 = 36%) or 40% (79/505 = 16%). With these consequences in mind, we analyze responses to punishment.

Participants who returned 50% or more of the 210 points they received favored their partner, the sham truster, over themselves; they were never punished for this decision. Of these participants, 34% wanted to switch partners (95/278). The 227 participants who returned 40% or less favored themselves over their partner. Of those who were *not* punished, 31% wanted to switch partners (35/113)—comparable to the 34% found for those who returned 50% or more. So, in the absence of punishment, about one-third of participants decided to switch partners.

**Fig 3:**
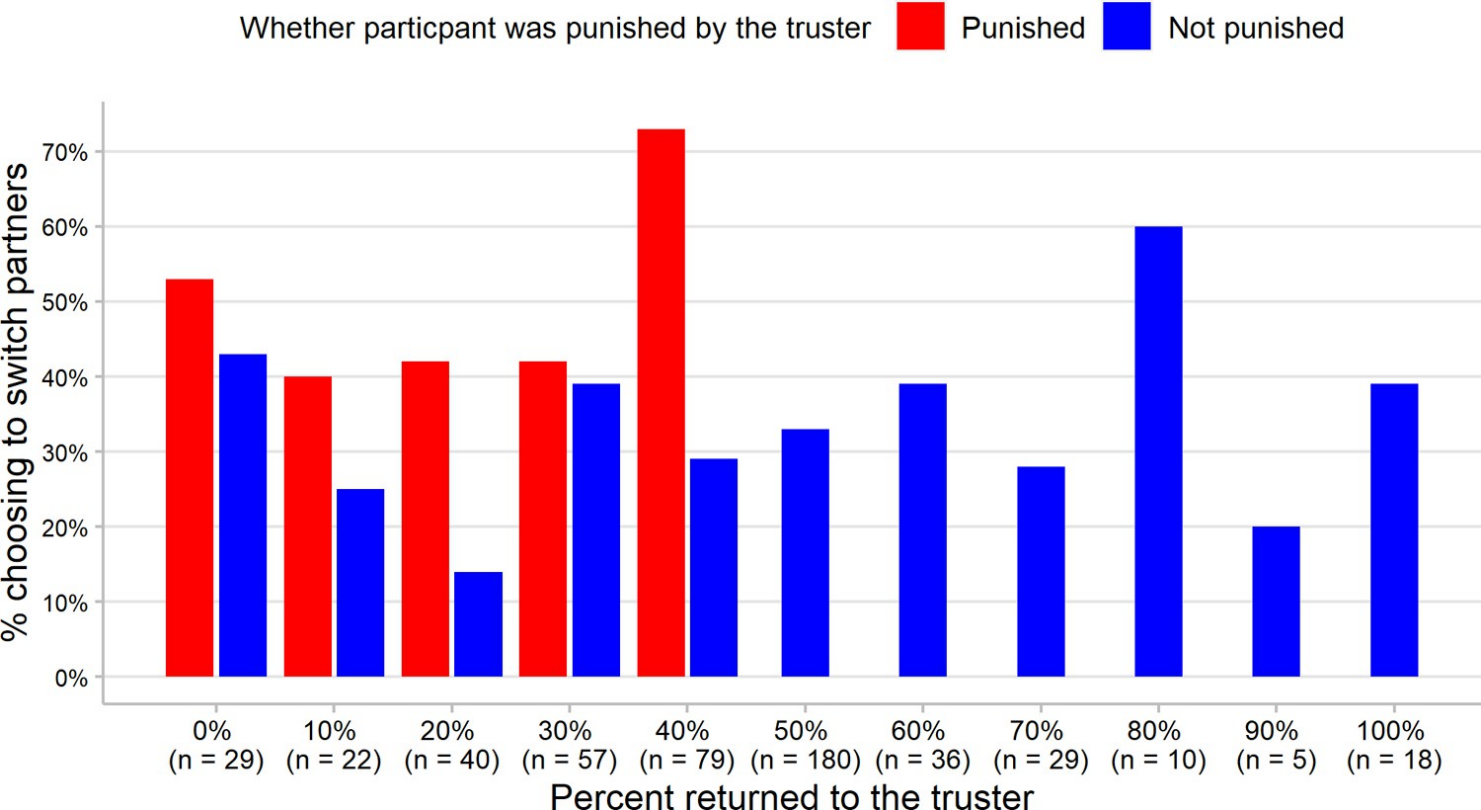
Probability of switching partners as a function of how much the participant returned and whether the participant was punished. The y-axis shows the percentage of participants who decided to switch partners in the High Partner Choice condition. The x-axis shows what percent of 210 points the participant (responder) returned to the partner (truster). (How many individuals returned each amount is shown in parentheses.) Red bars: participants whose partner punished them by deducting 60 points; blue bars: participants who were not punished by their partner.

We next examine the effect of being punished on the 227 participants who returned 40% or less. These participants favored themselves over the sham truster, but to different degrees. Fig 3 shows how many participants returned from 0 to 100%, and the probability that they wanted to switch partners as a function of being punished by their current partner.

For the participants who returned 40% or less (*n* = 227), the probability of switching was higher for those punished than for those who were not punished: 53.5% versus 31% (61/114 vs. 35/113; *Z* = 3.44, *p* = .0003). They were 3.35 times more likely to switch when the truster punished them than not, controlling for all other predictors (OR = 3.35, 95% CI = [1.84, 6.27]).

What about unjust punishment? Those who returned 30% or less are clearly defectors, but is returning 40% a failure to reciprocate? These responders satisfied both concepts of reciprocity: 40% provides a positive payoff to both parties (114 points for truster, 126 points for responder) with the smallest deviation from equality—a 12-point difference. Returning 50%—105 points—is an equal division of the points the responder *received* but, because the truster kept 30 points of the initial endowment, it results in a larger departure from equality: a 30-point difference (135 points for truster, 105 points for responder). Do those who returned 40%—a positive payoff with almost equal outcomes—feel wronged by being punished?

Of the 79 participants who returned 40%, 73% of those who were punished wanted to switch partners (27/37), compared to 33% of those who were not punished (12/42). Controlling for other predictors, those who were punished for returning 40% were almost *ten times* more likely to switch partners than those who were not punished (OR = 9.58, 95% CI = [2.82, 40.81]). By contrast, being punished had no significant effect on the true defectors—those who inflicted a negative payoff by returning 30% or less (*n* = 148) (OR = 1.99, 95% CI = [0.94, 4.33]). (See S4 Appendix)

#### 3.1.2 Participants were more likely to stay with responders who reciprocated their trust

As truster, the participant could send 0 ≤ P ≤ 100 points to the responder. Sending P > 0 points creates 3P points, making cooperation for mutual benefit possible. The truster’s payoff is 100 –P +.X(3P). Both benefit if the (sham) responder reciprocates by returning 50% of the 3P points: The truster gains a positive payoff because 100 –P + .5(3P) = 100 + .5P > 100. Returning 20% ensures a loss for the truster: 100 –P + .2(3P) = 100 - .4P < 100. This is a failure to reciprocate, indeed a defection (see above Section 3.1.1). Participants were far more likely to leave defectors—partners who returned only 20% of 3P—than reciprocators (those who returned 50% of 3P): OR = 4.45 (95% CI = [2.91, 6.89]). Reciprocation by the responder greatly increased the probability that the participant wanted to continue their partnership.

#### 3.1.3 Did participants who punished their partner want to remain in that relationship?

Switching partners defeats the purpose of punishing your *current* partner, if the function of punishing is to elicit more cooperation from a partner you plan to stay with [2]. Yet those who punished their partner were not more likely to remain in that relationship; indeed, controlling for other predictors, the more points participants paid to punish the partner, the more likely they were to leave the punished partner (*Amount paid to punish the responder*: OR = 1.02; 95% CI = [1.01, 1.03]).

#### 3.1.4 What else affected partner switching?

The more points participants entrusted to their partner, the more likely they were to want a new partner, although the effect was very small (*Trust*: OR = 1.01; 95% CI = [1.00, 1.02]). Also, American participants were less likely to switch partners than Japanese participants (*Society*: OR = 0.64; 95% CI = [0.42, 0.97]). How much participants returned to the truster was unrelated to their probability of switching partners (*Reciprocation by the participant*: OR = 1.00; 95% CI = [0.99, 1.01]).

#### 3.1.5 Partner switching summary

Having been punished was the second largest predictor of the decision to leave one’s partner in this study. Participants were less likely to stay with responders who defected—the largest predictor—and, all else equal, Americans were more likely to stay than Japanese participants. This implies that one’s reputation as a cooperator affects the probability of keeping a partner: Reciprocation increases that probability and punishing decreases it. Participants’ assumptions about relational mobility in their society did not predict their own decision to switch; their partner’s behavior did.

### 3.2 Inflicting punishment

#### 3.2.1 Did participants pay to punish?

When participants were the truster, they could punish their partner’s response, whether the partner had returned 50% or 20% of 3P points. Twenty-seven percent of participants chose to inflict punishment (282/1039); 78% of these individuals were punishing defectors—those who returned 20% of 3P points (219/282). When the partner had defected, 44% of participants paid to punish the defection (219/496). Only 12% punished partners who had returned 50% of 3P points (63/543). (Trusters who punished reciprocators risked about 15 fewer points as truster than those who did not [P = ~45 vs. ~62 points] and were more likely to have been punished in round 1 [46% vs. 16% punished].)

Participants could pay 0–50 points (in 10-point increments) to punish their partner’s response, whether the partner had defected or reciprocated. The mean of amount of punishment inflicted was 8.15 points (*SD* 15.61; range 0–50; median = 0). As expected, the mean was higher in response to defection than reciprocation: 13.59 (*SD* 18.63) vs. 3.19 (*SD* 9.91), *t* (738.69) = 11.09, *p* = 10^−16^).

When a responder defects, trusters who risked more suffer greater losses; many theories predict that greater losses will up-regulate motivations to impose negative sanctions—whether these involve withdrawing benefits or inflicting costs [62]. *Trust*—the number of points the truster risked—was indeed correlated with the desire to withdraw benefits by leaving a defecting partner: *r* (242) = .31 (*p* = 10^−6^). Inflicting costs showed the same pattern and effect size: Participants who sent more points as truster paid more to punish a defecting partner: *r* (494) = .31 (*p* = 10^−12^). For this reason, the analyses that follow control for both *Trust*, that is, P, the number of points the participant risked, and defection by the sham responder.

#### 3.2.2 Did participants punish less when they thought others can exercise partner choice?

The results on partner switching (section 3.1) showed that being punished drives partners away: Inflicting punishment decreases the probability that others will choose to partner with you. This is a liability when other people can exercise partner choice. We therefore predicted that the perception that others can easily switch partners will down-regulate motivations to punish. To test this prediction, we assessed the amount of punishment delivered as a function of relational mobility—an estimate of partner choice in one’s local social ecology—and verbal cues about partner choice in the immediate situation, which were delivered with the instructions. We also controlled for society: US versus Japan.

To determine which of these variables predicted amount of punishment delivered (*Amount paid to punish the responder*: 0–50), we conducted multiple linear regression with the glm function in R [60]. The predictors examined were condition (High Partner Choice = 1, Low Partner Choice = 0), society (US = 1, Japan = 0), and participants’ perceived relational mobility (*RM others* and *RM self*). These analyses controlled for whether the responder reciprocated or defected on the participant (*Defection by the responder*: 50% returned = 0, 20% returned = 1) and how many points participants entrusted to their partner (*Trust*: 0–100).

We also entered interactions between the predictors and, with stepwise selection, determined the best model (using the step function in R [60]). Based on AIC scores, the interactions and *RM self* were removed from the model. All continuous variables were centered by subtracting the mean to avoid multicollinearity issues [61] (resulting VIF values < 1.3). See S3 Appendix for full models with unstandardized coefficients, associated confidence intervals, and adjusted R^2^.

In determining responses, directly experiencing how a specific partner behaves should have greater weight than any social ecological variable. We did indeed find carry-over effects: The partner’s behavior in the first round affected participants’ responses in the second round (whether the participant was truster or responder in round 1). To see whether social ecology variables predict punishment *in the absence of a prior history* with the current partner, we analyzed *Amount paid to punish the responder* by those who played the truster role first (*n* = 509). (Of these, *n* = 238 experienced a responder who defected.)

#### 3.2.3 Did telling people they could switch partners decrease their motivation to punish?

No. There was no effect of Low vs. High Partner Choice condition on how much participants paid to punish (β = -0.002, *p* = .96; *n* = 509). Telling them in advance whether they will play all rounds with their current partner (Low Partner Choice) or have the opportunity to switch partners after round 2 (High Partner Choice condition) did not influence their punishment decisions, even if we restrict the analysis to the participants who experienced defection (β = -0.03, *p* = .68; *n* = 238). Note that this is not because participants were insensitive to the partner choice condition: We found that the condition did affect *Reciprocation by the participant* (DV1) (see Section 3.3.1).

#### 3.2.4 Did how much people punished differ by society (US vs. Japan)?

Yes. All else equal, American participants paid more to punish their responder than Japanese participants did (β = 0.13, *p* = .003, *n* = 487). The effect of society was similar (but not significant) when the analysis is restricted to the 238 participants whose responder defected (β = 0.11, *p* = .107).

#### 3.2.5 Did perceptions of relational mobility in their local social ecology affect how much people paid to punish?

**Fig 4:**
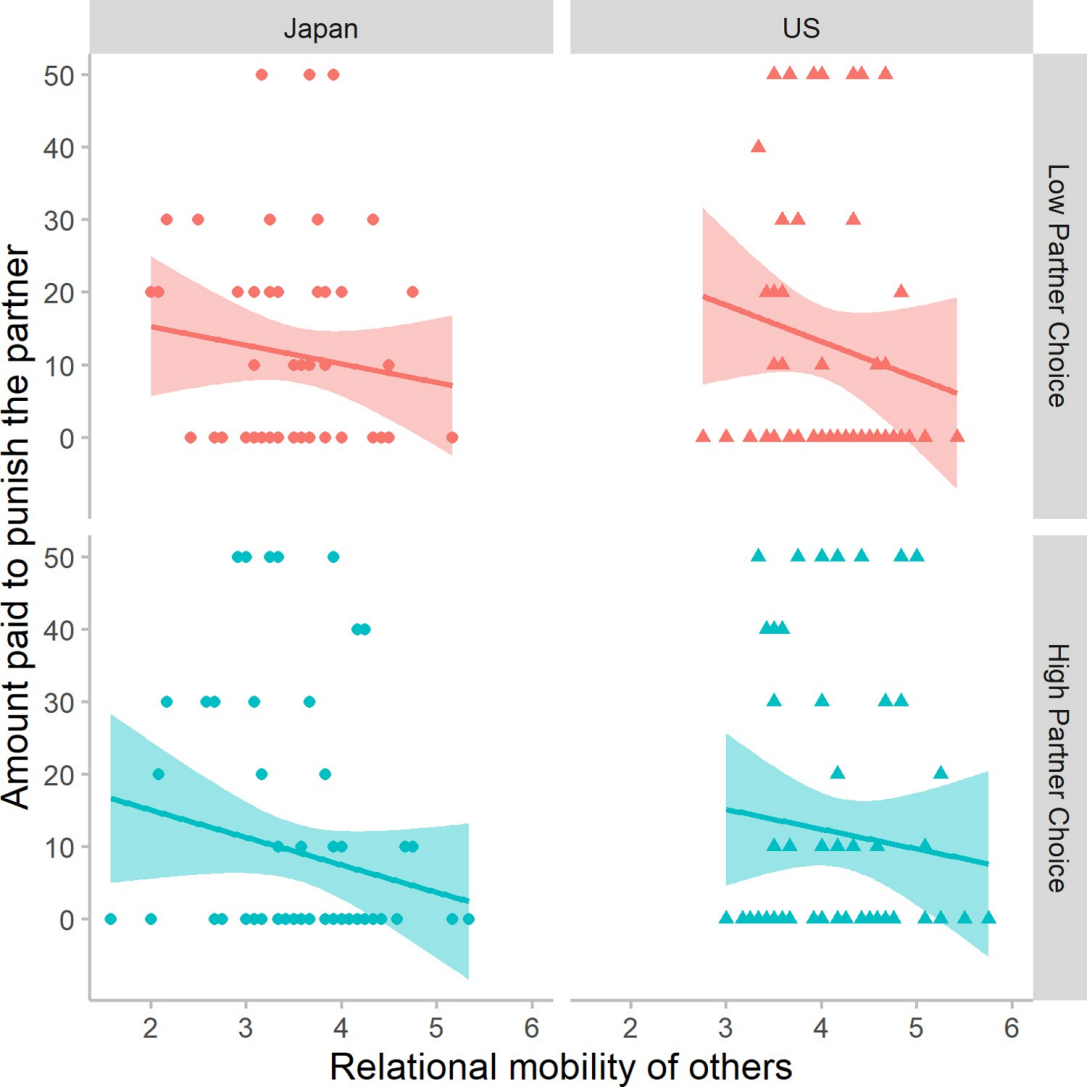
The effect of perceived relational mobility of others on punishment. Perceptions of other people’s relational mobility (*RM others*) was negatively associated with how much the participant paid to punish their partner who defected. The more they thought others could exercise partner choice, the less they punished the partner.

Yes: The higher their *RM others* score, the less participants paid to punish their partners (β = -0.14, *p* = .002, *n* = 487; when analyzing only those whose responder defected, β = -0.20, *p* = .004, *n* = 238). That is, the more opportunities they think others have to form new relationships, the less participants punished their partners. Equivalently: Those who assume the average person in their social ecology has fewer outside options inflicted more punishment. Fig 4 illustrates that this negative association holds, regardless of condition and society.

### 3.3 What predicts reciprocation?

*Reciprocation by the participant* refers to the percent of 3P points that the participant returned to the (sham) truster (3P = 210). The mean returned was 43.72% of 3P (*SD* = 21.43) and the median was 50%; that is, most responders gave their partner a positive payoff by returning 40% to 100%. For those who responded in the first round (*n =* 530)—before experiencing any punishment—71% gave their partner a positive payoff (67% of Americans, 76% of Japanese).

The more partner choice other people can exercise, the more motivated one should be to be seen as a good cooperative partner. This led us to predict that motivations to reciprocate would be up-regulated by perceptions that other people can easily switch partners. To test this prediction, we conducted a multiple regression for *Reciprocation* in the same way as for *Amount paid to punish the responder* (predictors: condition, society, *RM others*, *RM self*, and their interaction terms). *RM self* was removed from the model based on AIC scores. After centering the continuous variables (see Section 3.2.2), we found no evidence of multicollinearity (VIF values < 4.4). As before, we only analyzed *Reciprocation* by participants who played the responder role first, to avoid carry-over effects from round 1 (*n =* 530; 270 Americans and 260 Japanese). See S3 Appendix for full models.

#### 3.3.1 Did telling people they could switch partners increase reciprocation?

Even though we found no effect of condition on participants’ punishment behaviors, it did significantly influence their motivation to reciprocate. Telling participants in advance whether they would play all rounds with their current partner (Low partner choice) or have the opportunity to switch partners after round 2 (High Partner Choice condition) had no main effect on *Reciprocation by the participant* (β = 0.10, *p* = .118), but it interacted with the other predictors.

There was a 2-way interaction between condition and society (β = -0.18, *p* = .023) and a 3-way interaction between condition, society, and *RM others* (β = 0.24, *p* = .006). These significant interaction effects indicate that participants did detect, register, and respond to the verbal cue about the possibility of partner choice in their immediate situation. To examine these interactions, which all involve society, we ran the same regression model for each society separately (see Section 3.3.4).

#### 3.3.2 Did society (US vs. Japan) affect reciprocation?

Yes. Japanese participants returned a larger percentage of the 3P points sent by the (sham) truster than Americans did (β = -0.13, *p* = .036). The difference between societies was carried by the extremes. Americans were more likely than Japanese to defect by returning 30% or less (33% vs. 24%, *Z* = 2.64, *p* = .008 [90/270 vs. 63/260]) and less likely to reciprocate generously by returning 60% or more (13% vs. 30%, *Z* = -4.74, *p* = 10^−5^ [36/270 vs. 78/260]). There was no difference in how likely American and Japanese participants were to return 40% (16% vs. 15% [43/270 vs. 40/260]) or 50% (38% vs. 30% [101/270 vs. 79/260]).

There was also a 2-way interaction between society and *RM others* (β = -0.24, *p* = .004) (see Section 3.3.4).

#### 3.3.3 Did perceptions of relational mobility in their local social ecology affect participants’ motivations to reciprocate?

**Fig 5:**
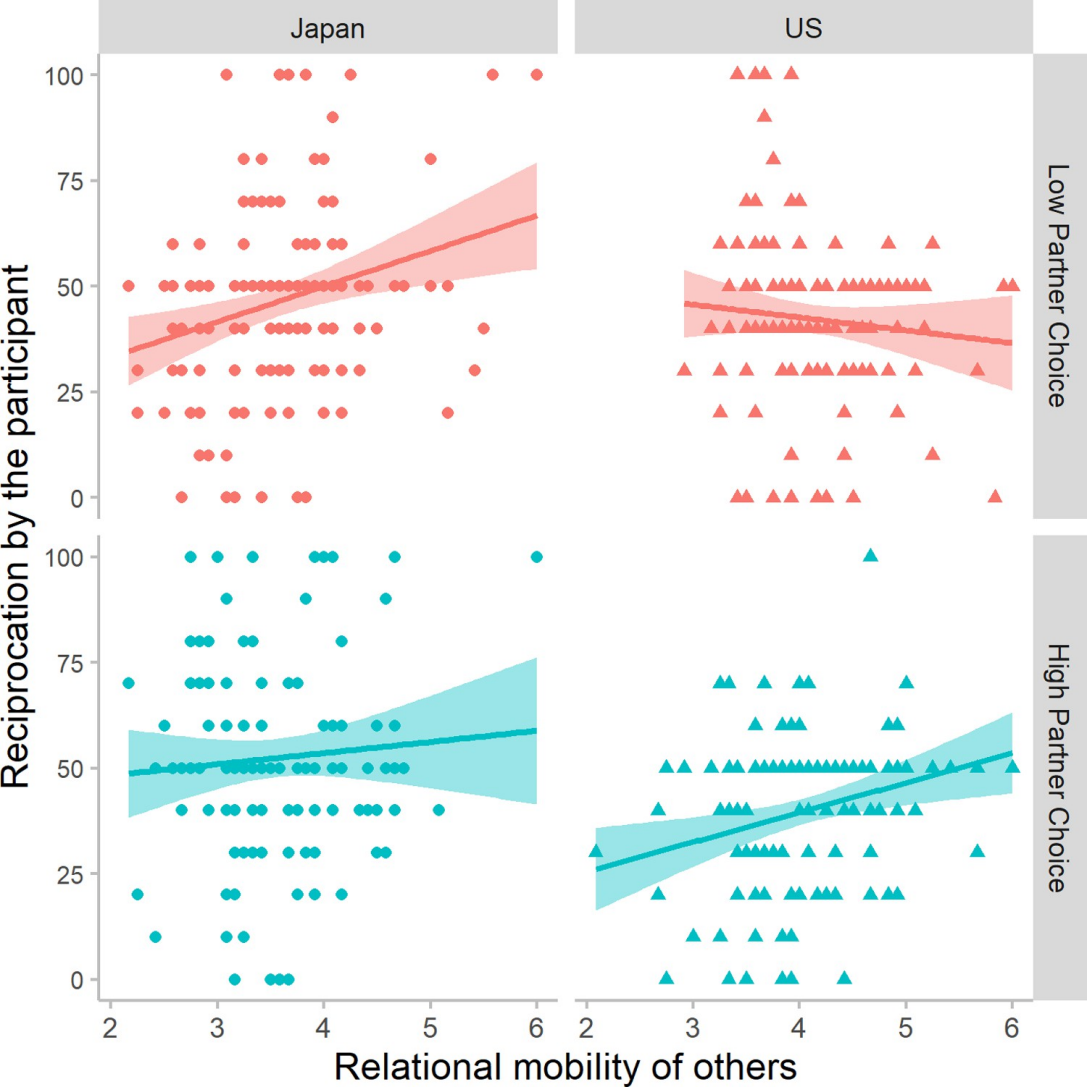
The effect of perceived relational mobility of others on reciprocation. Perceptions of other people’s relational mobility (*RM others*) was positively associated with the percentage of points the participant returned to the partner (0–100%). The more they thought others could exercise partner choice, the more they reciprocated.

Yes. Participants’ motivation to reciprocate their partner’s trust was up-regulated by their perceptions of relational mobility in their society (*RM others*: β = 0.27, *p* = .0008). The more opportunities they thought people in their social ecology have to leave unsatisfying relationships for better ones, the larger the percentage of 3P points they returned as responders (i.e., those who thought others have fewer opportunities to change relationship partners reciprocated less). Fig 5 illustrates that this positive association generally holds, regardless of society and condition, except for American participants in the Low Partner Choice condition.

#### 3.3.4 How did condition and perceived relational mobility interact in each society?

The patterns shown in Fig 5 suggest that the effect of perceived relational mobility might differ across conditions and societies. Indeed, there was a 3-way interaction between condition, society, and *RM others* (see Section 3.3.1). To examine this 3-way interaction, below we analyze the interaction between condition and *RM others* separately in Japan and the US.

Japanese participants (*n* = 260) up-regulated their motivation to reciprocate with their estimate of other people’s relational mobility (*RM others*: β = 0.25, *p* = .002); the effect size is about the same as when both societies were analyzed together. In Japan there was no significant main effect of condition (β = 0.10, *p* = .15) or interaction between condition and *RM others* (β = -0.11, *p* =. 175).

For American participants (*n* = 270), there was no main effect of *RM others* (β = -0.10, *p* = .273), but there was an interaction (β = 0.24, *p* = .008) between this variable and condition—whether they were told that they would play all rounds with the same partner or have the opportunity to switch after round 2. When told they would have the opportunity to switch partners, American’s perceptions of relational mobility regulated reciprocation, with the same effect size as found in Japan (High Partner Choice condition, *RM others* predicting *Reciprocation by the participant*: β = 0.25, *p* = .004, *n* = 128). This relationship was absent when Americans were told they would always interact with the same partner (Low Partner Choice condition, *RM others* predicting *Reciprocation by the participant*: β = -0.09, *p* = .306, *n* = 142). This is not because Americans treated their partners poorly when the situation precluded partner choice: Overall levels of reciprocation were similar across both conditions—42% (Low) vs. 40% (High)—even when controlling for *RM others* (β = -0.12, *p* = .07).

If relational mobility represents the prior probability that people can find new relationship partners in their local social ecology, then Americans updated that prior based on verbal cues regarding the immediate situation, but Japanese participants did not.

### 3.4 Are qualities of the person used to update priors based on social ecology?

Priors based on social ecology are most relevant when you have no other information about a new partner. This prior should be most strongly updated by learning what your new partner is like [63], with their actual behavior toward you a good cue to how they treat strangers. All else equal, a good cooperator will have more outside options than a defector, so efforts to retain your partner should increase with evidence that this partner is a valuable cooperator.

Consistent with this view, the partner’s behavior in round 1 influenced their behavior in round 2, with little or no remaining effect of social ecology. For example, reciprocation in round 2—the percent of 210 points that participants returned—reflected reciprocation vs. defection by the sham partner in round 1: They returned much more to sham partners who had reciprocated instead of defecting in round 1 (47.1% vs. 36.8%, *t* (457.72) = 5.49, *p* = 10^−8^). Controlling for all other factors, defection by the sham partner in round 1 predicted their reciprocation in round 2 (β = -.23, *p* = 10^−7^), but not their perceptions of relational mobility (controlling for round 1 behaviors, *RM others*: β = .06, *p* = .345). Similarly, when the partner defected in round 2, relational mobility had no influence on whether participants punished them (β = -.11, *p* = .092, controlling for other factors).

Participants who returned 40% or less in round 1 had a 50% chance of being punished. When these participants were trusters in round 2, relational mobility had no effect on how many points they risked, but their partner’s round 1 behavior did. Participants who had not been punished in round 1 risked more points in round 2 than those who had been punished (53.60 vs. 40.16, *t* (230.91) = 3.13, *p* = .002; controlling for other factors, *Punishment received* in round 1: β = -.22, *p* = .0002). This effect was strongest in those who were punished for returning 40% in round 1 (β = -.41, *p* = .0001). Those who were punished in round 1 also paid more to punish in round 2 than those who were not (12.05 vs. 6.49 points, *t* (233.79) = 2.76, *p* = .006; controlling for other factors, β = .23, *p* = .0001). That is, many engaged in retaliatory punishment.

In round 2, higher relational mobility had just one effect: Americans who believe people in their social ecology can easily switch partners were less likely to punish a partner who had reciprocated their trust (i.e., it decreased anti-social punishment in round 2; β = -.31, *p* = .012, controlling for condition). (See S5 Appendix for the full regression models.)

## 4 Discussion

### 4.1 Evidence that motivational systems are designed for social ecologies with varying levels of partner choice

Ancestral variation in the availability of cooperative partners would have favored the evolution of motivational systems that treat partner choice as a continuous variable. Motivations to keep valuable cooperative partners and abandon unrewarding ones should be up-regulated in response to the perception that others can easily switch partners.

Here we tested the hypothesis that an individual’s motivations to reciprocate and punish are calibrated by that person’s estimate of the degree to which others in their local social ecology can exercise partner choice. This estimate is captured by measures of *relational mobility* [50]. The higher an individual’s relational mobility score, the more opportunities they believe others have to leave unsatisfying relationships for better ones.

We assessed motivations to trust, reciprocate, defect, punish, and switch partners by allowing people to cooperate for mutual benefit with a new individual. The results showed that motivations to reciprocate and punish tracked participants’ perceptions of relational mobility. The more partner choice they thought others in their social ecology could exercise, the more they reciprocated their partner’s trust and the less they paid to punish their partner—even when that partner had defected.

Providing incentives for desirable partners to stay in the relationship is the proposed function of these motivational calibrations. If that is correct, then people who have the opportunity to switch partners will be more likely to stay with a partner who reciprocates their trust and more likely to leave one who punishes them. After two rounds, half the participants were asked if they wanted to keep their current partner or switch to someone new. Holding all else equal, having been defected on more than quadrupled the odds that they wanted to switch and having been punished tripled the odds they would choose to leave. These were the two biggest independent predictors of switching decisions. The desire to leave a partner who punished was especially strong for participants who returned 40%—a response that creates a positive payoff for both parties that is almost equal. These individuals were almost 10 times more likely to want a new partner.

#### 4.1.1 Are priors about social ecology updated by information about the situation or the person?

Perceptions of relational mobility are based on a huge database of experiences in a local social ecology—sometimes a lifetime’s worth. For this reason, we proposed that relational mobility serves as an estimate of the prior probability that others in one’s social ecology can exercise partner choice. It is a best guess before you learn what your partner is like—the situation participants faced in round 1.

If relational mobility in your social ecology is used to estimate a partner’s outside options when you know nothing else about that person, then its effect on cooperative motivations should be reduced (or eliminated) by data about that specific person’s value as a cooperative partner—to yourself and others. The evidence indicates that participants in both societies updated this prior based on first-hand knowledge of their partner’s willingness to cooperate and reluctance to punish. Once participants had experienced how their partner behaved in round 1, relational mobility no longer predicted how much they trusted, reciprocated, or punished in round 2, in either the US or Japan. The behavior of the sham partner in round 1 (and, of course, in round 2) did predict their responses. The only behavior that relational mobility continued to influence was antisocial punishment. The belief others in your social ecology can easily switch partners tempered—but did not eliminate—antisocial punishment. (See S5 Appendix.)

The results suggest that estimates of partner choice based on social ecology are updated based on properties of the *person* with whom one is interacting. But are these estimates updated in response to cues about a temporary *situation* one is facing—ones unrelated to the partner’s value as a cooperator? It is not clear that they should be.

Delton et al. [35] examined the evolution of motivations to cooperate in Bayesian agents who knew the base rate of one-shot interactions in their population and updated this prior based on a cue about the immediate situation they were facing. The cue reflected the probability that they would never interact again with their current partner. These Bayesian agents evolved a strong disposition to cooperate *even when they rationally believed the interaction was one-shot*. Selection favored agents who behaved *as if* they would repeatedly interact with their current partner even when they knew this was unlikely. Agent-based models also show that meeting a new individual once was a good cue that you will meet them again in ancestral social ecologies [64]. Every participant in our study was exposed to this ancestrally-reliable cue to a shadow of the future: They interacted with their partner for two rounds.

We did, however, provide a verbal cue relevant to partner choice in the temporary situation that they were facing. Half the participants were told they would be interacting with the same partner in every round (i.e., they were engaged in a repeated interaction with this person). The other half were told they could change partners after two rounds (i.e., their current partner can refuse to interact with them repeatedly). If this verbal cue is used to (temporarily) update their prior probability that a newly encountered person can exercise partner choice, their motivations to cooperate or punish might shift in response.

There was little evidence that participants in round 1 used this situational cue to update a prior that was based on their social ecology. Being told whether they would have the opportunity to switch partners had no effect on how much participants punished defections by their partner: Higher relational mobility in their local social ecology predicted less punishment, regardless of condition or society. The cue did have an effect on how much American participants reciprocated their partner’s trust, however. Although average levels of reciprocation were similar in both conditions, higher relational mobility predicted more reciprocation when Americans were told they and their partner could part ways after two rounds, but not when they were told that all of their interactions would be with the same partner.

Japanese participants did not respond to this cue at all: Their estimates of relational mobility predicted more reciprocation (and less punishment) to the same extent in both conditions. That is, there was no evidence that people in Japan updated their prior hypothesis about relational mobility based on the situational cue we provided. If they did, the change was too small to influence their willingness to reciprocate or punish.

If this result generalizes to other cues about a temporary situation, it suggests that the benefits of opportunistic behavior in the short term were generally outweighed by the risk of losing a valuable, long-term cooperative partner.

### 4.2 What is the function of punishment in dyadic reciprocal cooperation?

What, if anything, is the adaptive function of motivations to pay a cost to punish a defecting partner? This was not a rare response: Of participants who were trusters in round 1, 44% punished when the responder defected. It is usually assumed that the function of punishing defectors is to elicit more cooperation from them in the future—especially when they do not have the option to change partners.

People who believe others in their social ecology have fewer options to switch partners did pay more to punish defectors: Low relational mobility scores predicted paying more to punish. But there was no evidence that punishment succeeded in eliciting greater cooperation from participants. Quite the contrary: Participants who were punished for returning 0–40% in round 1 did not respond by sending more points as truster in round 2. Indeed, they returned fewer points as truster (β = -.22, *p* = .0002), and this effect was particularly pronounced for those who had provided a positive payoff by returning 40% in round 1, β = -.41, *p* = .0001 (vs. β = -.12, *p* = .099 for those who provided a negative payoff in round 1; see S5 Appendix). Moreover, those who were punished in round 1 were more likely to retaliate by punishing their partner in round 2 (S5 Appendix; for similar results, see [65, 66]).

Not only did punishment fail to elicit more cooperation from punished partners, but it also drove them away. When partner switching was possible, having been punished was one of the biggest independent predictors of wanting to change partners. Driving away defectors might be a function of punishment, of course—when they were not punished, ~70% of people who returned 0–40% wanted to remain with their accommodating partner (~68% of those who returned 0–30%; ~71% of those returning 40%). Although participants in this study could prevent future interactions at lower cost by simply deciding to switch after round 2, avoiding unrewarding partners may be more difficult in real life, especially when they want to continue cooperating with you.

Krasnow et al. [57] suggest that punishing defection signals a willingness to continue cooperating with your current partner, but on more favorable terms. Using a paradigm similar to the TGP, they found that participants who punished a defecting partner in the first round were 11 times more likely to cooperate than defect in the second one (switching was not an option). This pattern was not apparent in our study: Participants who punished a defecting partner did not return more in round 2 than those who did not (39.28% vs. 35.18%, *t* (226.99) = 1.41, *p* = .160), and they were not more likely to want to remain with their partner—indeed, the more points participants paid to punish the partner, the more—although slightly—they wanted to switch (OR = 1.02; 95% CI = [1.01, 1.03]). (Note, however, that a participant’s decision to stay did not ensure a continuing interaction in our study; the partner also had the option to leave, and punished ones were likely to do so.)

Our results showing that retaliatory punishment was common—~45% of those who were punished in round 1 retaliated in round 2—suggest an alternative explanation. In Krasnow et al. [57], participants who punished defectors in round 1 may have cooperated in round 2 to avoid (very costly) retaliatory punishment by their partner. Those who did not punish partners who succumbed to the temptation to cheat in round 1 may have assumed their partner would “reciprocate” by not punishing when them when they did the same in round 2.

Motivations to punish did not reflect the participant’s own commitment to stay in the relationship, but they were up-regulated by estimates that *partners* might have few outside options: Lower relational mobility in one’s social ecology did predict amount paid to punish defectors. The results are consistent with the hypothesis that motivations to punish evolved to deter bad treatment in the future by partners who do not seem to value your welfare [67]. Defecting now may be a reliable cue that this partner does not value your welfare sufficiently, and punishment was overwhelmingly directed at defectors. In ancestral social ecologies, partners who part ways now may nevertheless have to cooperate again in the future [64, 67, 68]. Punishment may have evolved as a warning, to deter bad treatment by defectors who may darken your door in the future.

### 4.3 Micro and macro effects of social ecology

We measured two variables regarding participants’ real-life social ecology of partner choice. First, we measured participants’ perceptions of their partner choice ecology with the relational mobility scale [50]. Second, we recruited participants from two societies in which average relational mobility scores are typically high (US) versus low (Japan). This lets us see whether behavior at the individual level scales up to explain differences between nations.

Within each society, the motivations of individuals were calibrated by their perceptions of other people’s relational mobility: the number of opportunities they believe that others have to form new relationships. Moreover, the pattern of calibration was universal: Within each society, higher relational mobility scores predicted more reciprocation and less punishment. Individual-level effects tracked individual perceptions of the local social ecology.

What about group-level differences? The concept of relational mobility was built from Yamagishi’s seminal work on general trust: a cognitive bias to assume that newly encountered people will treat you with benevolence rather than exploitation [69, 70]. *General trust* varies across nations; scores on the standard survey measure are higher in the US than Japan, for example. Where general trust is higher, people are more willing to risk cooperating with strangers who could, if untrustworthy, profit at their expense. The benefit of trusting strangers is that it allows people to discover better cooperative partners, giving them more outside options. The resulting increase relational mobility then tempers the risk of trusting strangers: The threat that a good partner will leave for a better outside option can deter exploitive behavior and increase benevolence.

With this in mind, we compared average behavior in the US and Japan. As in other studies, perceptions of relational mobility were higher in the US than Japan (*RM others*: 4.12 vs. 3.57, *t* (1028.2) = 13.76, *p* = 10^−16^; *RM self*: 4.20 vs. 3.37, *t* (1030.8) = 18.71, *p* = 10^−16^). That is, the average American believes others have more outside options than the average person from Japan does. Moreover, as Yamagishi’s view of general trust predicts, when participants had no prior experiences with their partners, American trusters risked more points on a stranger than Japanese participants did (*Trust*: 59 vs. 50.6, *t* (502.55) = 2.9, *p* = .004). And trusting strangers usually paid off: Most responders delivered a positive payoff in both societies (US 67%, JP 76%).

Did the perception that others have more outside options lead the average American to reciprocate more and punish less than the average person from Japan? No. Not only did Americans return less, on average, than Japanese participants, but more of them exploited their partner’s trust by delivering a negative payoff (US 33% vs. JP 24%). Americans were also more punitive, not less: They paid more to punish their partners, even when controlling for all other factors (including whether their partner defected). And, despite less reciprocation and more punishment at the macro-level, Americans were more likely to stay with their partner than Japanese participants (all else equal).

Within each society, individual differences in reciprocation and punishment were associated with individual differences in perceptions of relational mobility, but this did not translate into group-level differences between the US and Japan. Assuming that individual differences fully explain group-level differences is called the *ecological fallacy* [71–73]. The data clearly show that the micro-level effect of individuals’ perceptions of relational mobility and the macro-level effect of society were independent of one another. The individual-level psychological calibrations and the group-level differences between nations coexist, rather than one producing the other.

Features of the social ecology other than relational mobility could be responsible for the differences in group-level calibrations between the US and Japan (see e.g., [56, 74]). That Japanese participants were less punitive than Americans is contrary to findings that Japan (or East Asian countries in general) has “tighter” norms than the US which, when broken, elicit great censure [75, 76], but perhaps consistent with studies showing greater motivations to avoid rejection in people in Japan than the US [74]. Our data cannot speak to these explanations of the group-level differences we found.

### 4.4 Limitations and future directions

Motivations responded when participants learned how the partner treats them, but the partner switching instructions influenced Americans only (and not much at that). This could be because repeated interactions—with interruptions between—were common ancestrally, making long-run estimates of social ecology a more reliable basis for calibration than cues about a fleeting situation. The other possibility is that a cue delivered online was too divorced from real life, devoid of psychophysical cues typical of social isolation versus community. Future studies might enhance the salience of the situational cue, perhaps by including visual displays showing many versus few alternative partners (avatars or faces), or by giving participants prior experiences of a desirable partner leaving for a better one or an unrewarding partner staying.

A person with fewer outside options than others in their local ecology may feel they need to reciprocate more and punish less. We did adapt the relational mobility scale to ask about the self; although self and other scores were correlated *r* (515) = .60 (*p* = 10^−16^) in the US and *r* (516) = .50 (*p* = 10^−16^) in Japan, we calculated whether *RM self* < *RM other* for each participant. In Japan, 67% of participants felt their outside options were worse than those of other people, compared to 44% in the US. And, in both countries, those who felt they have fewer outside options returned more points than those who felt their options were better than or equal to others, but the difference in points returned was not significant. A better measure in the future might be to ask, for each RM question, whether people feel they have more, the same, or fewer options than others in their society.

Dyadic cooperation may be affected by other aspects of the social ecology as well, such as how likely others will be to take advantage of you [69]. Punishment as a deterrent may be up-regulated in ecologies where the probability of being exploited are higher, as they were in the US in this study. Perceptions of these probabilities would be a fruitful variable to assess.

Lastly, our participants were from either the US or Japan, two populous, large-scale industrialized societies. Objectively speaking, most people in these countries are free to associate with anyone they like, and they are surrounded by strangers, each of whom is a potential new partner. It would be fruitful to extend the current line of research to smaller societies in which the actual—not only perceived—possibility of partner choice is more limited.

## 5 Conclusions

We demonstrate that estimates of partner choice in one’s local social ecology systematically regulate motivations to reciprocate, defect, and punish in dyadic cooperative interactions. The more opportunities participants thought others have to form new relationships, the more they reciprocated and the less they punished. The results suggest that the function of these motivational calibrations is to retain valuable cooperative partners when they have the option to leave: When given the choice, participants preferred to stay with partners who reciprocated and leave partners who punished them. The results support the hypothesis that motivational systems are designed to use estimates of the degree of partner choice in one’s local social ecology to functionally regulate cooperative behavior.

## Supporting information

S1 Appendix Instructions given to participants. (DOCX)

S2 Appendix Raw data. (XLSX)

S3 Appendix Full regression models. (DOCX)

S4 Appendix Full logistic regression models for the decision to switch partners given different reciprocation rates by the participant. (DOCX)

S5 Appendix Regression models for behaviors in round 2. (DOCX)

## References

[1] Trivers RL. The evolution of reciprocal altruism. Q Rev Biol. 1971;46: 35–57.

[2] Axelrod R, Hamilton WD. The evolution of cooperation. Science. 1981;211: 1390–1396. doi: 10.1126/science.7466396 7466396

[3] Hammerstein P, Noë R. Biological trade and markets. Philos Trans R Soc B Biol Sci. 2016;371. doi: 10.1098/rstb.2015.0101 26729940 PMC4760201

[4] Schweinfurth MK, Taborsky M. Rats play tit-for-tat instead of integrating social experience over multiple interactions. Proc R Soc B Biol Sci. 2020;287. doi: 10.1098/rspb.2019.2423 31937222 PMC7003459

[5] Dugatkin LA, Alfieri M. Tit-For-Tat in guppies (Poecilia reticulata): the relative nature of cooperation and defection during predator inspection. Evol Ecol. 1991;5: 300–309. doi: 10.1007/BF02214234

[6] Bshary R, Grutter AS. Asymmetric cheating opportunities and partner control in a cleaner fish mutualism. Anim Behav. 2002;63: 547–555. doi: 10.1006/anbe.2001.1937

[7] Schuessler R. Exit Threats and Cooperation under Anonymity. J Conflict Resolut. 1989;33: 728–749. doi: 10.1177/0022002789033004007

[8] Hayashi N. From tit-for-tat to out-for-tat. Scociological Theory and Methods. 1993;8: 19–32.

[9] Aktipis CA. Know when to walk away: Contingent movement and the evolution of cooperation. J Theor Biol. 2004;231: 249–260. doi: 10.1016/j.jtbi.2004.06.020 15380389

[10] Joyce D, Kennison J, Densmore O, Guerin S, Barr S, Charles E, et al. My way or the highway: A more naturalistic model of altruism tested in an iterative prisoners’ dilemma. Jasss. 2006;9: 79–92.

[11] Izquierdo SS, Izquierdo LR, Vega-Redondo F. The option to leave: Conditional dissociation in the evolution of cooperation. J Theor Biol. 2010;267: 76–84. doi: 10.1016/j.jtbi.2010.07.039 20688083

[12] Li WJ, Jiang LL, Perc M. A limited mobility of minorities facilitates cooperation in social dilemmas. Appl Math Comput. 2021;391. doi: 10.1016/j.amc.2020.125705

[13] Axelrod R. The evolution of cooperation. New York: Basic Books; 1984.

[14] Yamagishi T, Hayashi N, Jin N. Prisoner’s dilemma networks: Selection strategy versus action strategy. In: Schulz U, Albers W, Mueller U, editors. Social Dilemmas and Cooperation. Berlin: Springer-Verlag; 1994. pp. 233–250. doi: 10.1007/978-3-642-78860-4

[15] Bull JJ, Rice WR. Distinguishing mechanisms for the evolution of co-operation. J Theor Biol. 1991;149: 63–74. doi: 10.1016/s0022-5193(05)80072-4 1881147

[16] Noë R. A veto game played by baboons: a challenge to the use of the Prisoner’s Dilemma as a paradigm for reciprocity and cooperation. Anim Behav. 1990;39: 78–90.

[17] Leimar O, Hammerstein P. Evolution of cooperation through indirect reciprocity. Proc R Soc B Biol Sci. 2001;268: 745–753. doi: 10.1098/rspb.2000.1573 11321064 PMC1088665

[18] Ohtsuki H, Iwasa Y. The leading eight: Social norms that can maintain cooperation by indirect reciprocity. J Theor Biol. 2006;239: 435–444. doi: 10.1016/j.jtbi.2005.08.008 16174521

[19] Nowak MA, Sigmund K. Evolution of indirect reciprocity by image scoring. Nature. 1998;393: 573–577. doi: 10.1038/31225 9634232

[20] Bshary R, Grutter AS. Experimental evidence that partner choice is a driving force in the payoff distribution among cooperators or mutualists: The cleaner fish case. Ecol Lett. 2002;5: 130–136. doi: 10.1046/j.1461-0248.2002.00295.x

[21] Simms EL, Taylor DL, Povich J, Shefferson RP, Sachs JL, Urbina M, et al. An empirical test of partner choice mechanisms in a wild legume-rhizobium interaction. Proc R Soc B Biol Sci. 2006;273: 77–81. doi: 10.1098/rspb.2005.3292 16519238 PMC1560009

[22] Barclay P, Willer R. Partner choice creates competitive altruism in humans. Proc R Soc B Biol Sci. 2007;274: 749–753. doi: 10.1098/rspb.2006.0209 17255001 PMC2197220

[23] Barclay P, Raihani N. Partner choice versus punishment in human Prisoner’s Dilemmas. Evol Hum Behav. 2016. doi: 10.1016/j.evolhumbehav.2015.12.004

[24] Baumard N, André J-BB, Sperber D. A mutualistic approach to morality: The evolution of fairness by partner choice. Behav Brain Sci. 2013;36: 59–78. doi: 10.1017/S0140525X11002202 23445574

[25] Martin JW, Cushman F. To punish or to leave: Distinct cognitive processes underlie partner control and partner choice behaviors. PLoS One. 2015;10: e0125193. doi: 10.1371/journal.pone.0125193 25915550 PMC4411127

[26] Roberts G. Competitive altruism: From reciprocity to the handicap principle. Proc R Soc B Biol Sci. 1998;265: 427–431. doi: 10.1098/rspb.1998.0312

[27] Barclay P. Strategies for cooperation in biological markets, especially for humans. Evol Hum Behav. 2013;34: 164–175. doi: 10.1016/j.evolhumbehav.2013.02.002

[28] Quillien T. Evolution of conditional and unconditional commitment. J Theor Biol. 2020;492: 110204. doi: 10.1016/j.jtbi.2020.110204 32084497

[29] Eisenbruch AB, Grillot RL, Maestripieri D, Roney JR. Evidence of partner choice heuristics in a one-shot bargaining game. Evol Hum Behav. 2016;37: 429–439. doi: 10.1016/j.evolhumbehav.2016.04.002

[30] Bradley A, Lawrence C, Ferguson E. Does observability affect prosociality? Proc R Soc B Biol Sci. 2018;285. doi: 10.1098/rspb.2018.0116 29593114 PMC5897647

[31] Barclay P. Trustworthiness and competitive altruism can also solve the “tragedy of the commons.” Evol Hum Behav. 2004;25: 209–220. doi: 10.1016/j.evolhumbehav.2004.04.002

[32] Sylwester K, Roberts G. Cooperators benefit through reputation-based partner choice in economic games. Biol Lett. 2010;6: 659–662. doi: 10.1098/rsbl.2010.0209 20410026 PMC2936156

[33] Sylwester K, Roberts G. Reputation-based partner choice is an effective alternative to indirect reciprocity in solving social dilemmas. Evol Hum Behav. 2013;34: 201–206. doi: 10.1016/j.evolhumbehav.2012.11.009

[34] Delton AW, Cosmides L, Guemo M, Robertson TE, Tooby J. The Psychosemantics of Free Riding: Dissecting the Architecture of Moral Concept. J Pers Soc Psychol. 2012;102: 1252–1270. doi: 10.1037/a0027026 22268815 PMC3365621

[35] Delton AW, Krasnow MM, Cosmides L, Tooby J. Evolution of direct reciprocity under uncertainty can explain human generosity in one-shot encounters. Proc Natl Acad Sci. 2011;108: 13335–13340. doi: 10.1073/pnas.1102131108 21788489 PMC3156224

[36] Raihani NJ, Bshary R. The reputation of punishers. Trends Ecol Evol. 2015;30: 98–103. doi: 10.1016/j.tree.2014.12.003 25577128

[37] Horita Y. Punishers May Be Chosen as Providers But Not as Recipients. Lett Evol Behav Sci. 2010;1: 6–9. doi: 10.5178/lebs.2010.2

[38] Ozono H, Watabe M. Reputational benefit of punishment: Comparison among the punisher, rewarder, and non-sanctioner. Lett Evol Behav Sci. 2012;3: 21–24. doi: 10.5178/lebs.2012.22

[39] Dhaliwal N, Patil I, Cushman F. Reputational and cooperative benefits of third-party compensation. Organ Behav Hum Decis Process. 2021;164: 27–51. doi: 10.1016/j.obhdp.2021.01.003

[40] Przepiorka W, Liebe U. Generosity is a sign of trustworthiness-the punishment of selfishness is not. Evol Hum Behav. 2016;37: 255–262. doi: 10.1016/j.evolhumbehav.2015.12.003

[41] Sugiyama LS. Illness, Injury, and Disability among Shiwiar Forager-Horticulturalists: Implications of Health-Risk Buffering for the Evolution of Human Life History. Am J Phys Anthropol. 2004;123: 371–389. doi: 10.1002/ajpa.10325 15022365

[42] Gurven M, Hill K, Kaplan H, Hurtado A, Lyles R. Food transfers among Hiwi foragers of Venezuela: Tests of reciprocity. Hum Ecol. 2000;28: 171–218. doi: 10.1023/A:1007067919982

[43] Bliege Bird R, Scelza B, Bird DW, Smith EA. The hierarchy of virtue: mutualism, altruism and signaling in Martu women’s cooperative hunting. Evol Hum Behav. 2012;33: 64–78. doi: 10.1016/j.evolhumbehav.2011.05.007

[44] Kelly RL. Colonization of New Land by Hunter-Gatherers: Expectations and Implications Based on Ethnographic Data. In: Rockman M, Steele J, editors. The Colonization of Unfamiliar Landscapes: The Archaeology of Adaptation. Routledge; 2003. pp. 44–58.

[45] Ziegler M, Simon MH, Hall IR, Barker S, Stringer C, Zahn R. Development of Middle Stone Age innovation linked to rapid climate change. Nat Commun. 2013;4: 1905. doi: 10.1038/ncomms2897 23695699 PMC4354264

[46] Pietraszewski D, Curry OS, Petersen MB, Cosmides L, Tooby J. Constituents of political cognition: Race, party politics, and the alliance detection system. Cognition. 2015;140: 24–39. doi: 10.1016/j.cognition.2015.03.007 25867997

[47] Eisenbruch AB, Grillot RL, Roney JR. Why Be Generous? Tests of the Partner Choice and Threat Premium Models of Resource Division. Adapt Hum Behav Physiol. 2019;5: 274–296. doi: 10.1007/s40750-019-00117-0

[48] Debove S, Andre J-B, Baumard N, André J-B, Baumard N, Andre J-B, et al. Partner choice creates fairness in humans. Proc R Soc B Biol Sci. 2015;282: 20150392–20150392. doi: 10.1098/rspb.2015.0392 25972467 PMC4455807

[49] Yamagishi T, Jin N, Kiyonari T. Bounded Generalized Reciprocity: Ingroup boasting and ingroup favorism. Adv Gr Process. 1999;16: 161–197.

[50] Yuki M, Schug J, Horikawa H, Takemura K, Sato K, Yokota K, et al. Development of a scale to measure perceptions of relational mobility in society. CERSS Work Pap 75, Cent Exp Res Soc Sci Hokkaido Univ. 2007; 4–6.

[51] Thomson R, Yuki M, Talhelm T, Schug J, Kito M, Ayanian AH, et al. Relational mobility predicts social behaviors in 39 countries and is tied to historical farming and threat. Proc Natl Acad Sci. 2018;115: 7521–7526. doi: 10.1073/pnas.1713191115 29959208 PMC6055178

[52] Yuki M, Schug J. Psychological consequences of relational mobility. Curr Opin Psychol. 2020;32: 129–132. doi: 10.1016/j.copsyc.2019.07.029 31491705

[53] Oishi S, Schug J, Yuki M, Axt J. The psychology of residential and relational mobilities. In: Gelfand M, Chiu C, Hong Y, editors. Handbook of Advances in Culture and Psychology. Oxford: Oxford University Press; 2015. pp. 221–272.

[54] Schug J, Yuki M, Maddux W. Relational Mobility Explains Between- and Within-Culture Differences in Self-Disclosure to Close Friends. Psychol Sci. 2010;21: 1471–1478. doi: 10.1177/0956797610382786 20817913

[55] Komiya A, Ohtsubo Y, Nakanishi D, Oishi S. Gift-giving in romantic couples serves as a commitment signal: Relational mobility is associated with more frequent gift-giving. Evol Hum Behav. 2019;40: 160–166. doi: 10.1016/j.evolhumbehav.2018.10.003

[56] Yamagishi T, Hashimoto H, Schug J. Preferences versus strategies as explanations for culture-specific behavior. Psychol Sci. 2008;19: 579–584. doi: 10.1111/j.1467-9280.2008.02126.x 18578848

[57] Krasnow MM, Cosmides L, Pedersen EJ, Tooby J. What Are Punishment and Reputation for? PLoS One. 2012;7: e45662. doi: 10.1371/journal.pone.0045662 23049833 PMC3458883

[58] Herrmann B, Thöni C, Gächter S. Antisocial punishment across societies. Science. 2008. doi: 10.1126/science.1153808 18323447

[59] Shinada M, Yamagishi T, Ohmura Y. False friends are worse than bitter enemies: “Altruistic” punishment of in-group members. Evol Hum Behav. 2004;25: 379–393. doi: 10.1016/j.evolhumbehav.2004.08.001

[60] R Core Team. R: A Language and Environment for Statistical Computing. Vienna, Austria; 2020. Available: https://www.r-project.org/

[61] Robinson C, Schumacker R. Interaction effects: centering, variance inflation factor, and interpretation issues. Mult Linear Regres Viewpoints. 2009;35: 6–11.

[62] Petersen MB, Sell A, Tooby J, Cosmides L. To punish or repair? Evolutionary psychology and lay intuitions about modern criminal justice. Evol Hum Behav. 2012;33: 682–695. doi: 10.1016/j.evolhumbehav.2012.05.003 23412662 PMC3569042

[63] Jussim L. Social perception and social reality: A reflection-construction model. Psychol Rev. 1991;98: 54–73. doi: 10.1037//0033-295x.98.1.54

[64] Krasnow MM, Delton AW, Tooby J, Cosmides L. Meeting now suggests we will meet again: Implications for debates on the evolution of cooperation. Sci Rep. 2013;3: 1–8. doi: 10.1038/srep01747 23624437 PMC3638167

[65] Bone JE, Wallace B, Bshary R, Raihani NJ. The effect of power asymmetries on cooperation and punishment in a prisoner’s dilemma game. PLoS One. 2015;10: e0117183. doi: 10.1371/journal.pone.0117183 25629971 PMC4309618

[66] Bone JE, Wallace B, Bshary R, Raihani NJ. Power asymmetries and punishment in a prisoner’s dilemma with variable cooperative investment. PLoS One. 2016;11: e0155773. doi: 10.1371/journal.pone.0155773 27191958 PMC4871419

[67] Krasnow MM, Delton AW, Cosmides L, Tooby J. Looking Under the Hood of Third-Party Punishment Reveals Design for Personal Benefit. Psychol Sci. 2016;27: 405–418. doi: 10.1177/0956797615624469 26851057

[68] Smith KM, Larroucau T, Mabulla IA, Apicella CL. Hunter-Gatherers Maintain Assortativity in Cooperation despite High Levels of Residential Change and Mixing. Curr Biol. 2018; 3152–3157. doi: 10.1016/j.cub.2018.07.064 30245106

[69] Yamagishi T. Trust: the evolutionary game of mind and society. New York: Springer; 2011.

[70] Schug J, Yuki M, Horikawa H, Takemura K. Similarity attraction and actually selecting similar others: How cross-societal differences in relational mobility affect interpersonal similarity in Japan and the USA. Asian J Soc Psychol. 2009;12: 95–103. doi: 10.1111/j.1467-839X.2009.01277.x

[71] Thorndike EL. On the Fallacy of Imputing the Correlations Found for Groups to the Individuals or Smaller Groups Composing Them. Am J Psychol. 1939;52: 122. doi: 10.2307/1416673

[72] Pollet T V., Tybur JM, Frankenhuis WE, Rickard IJ. What can cross-cultural correlations teach us about human nature? Hum Nat. 2014;25: 410–429. doi: 10.1007/s12110-014-9206-3 25092392

[73] Brewer P, Venaik S. The Ecological Fallacy in National Culture Research. Organ Stud. 2014;35: 1063–1086. doi: 10.1177/0170840613517602

[74] Hashimoto H, Yamagishi T. Duality of independence and interdependence: An adaptationist perspective. Asian J Soc Psychol. 2016;19: 286ー297.

[75] Gelfand MJ, Raver JL, Nishii L, Leslie LM, Lun J, Lim BC, et al. Differences between tight and loose cultures: A 33-nation study. Science. 2011;332: 1100–1104. doi: 10.1126/science.1197754 21617077

[76] Wang CS, Leung AK -y. The Cultural Dynamics of Rewarding Honesty and Punishing Deception. Personal Soc Psychol Bull. 2010;36: 1529–1542. doi: 10.1177/0146167210385921 20947774

