# Supplementary material for: Motivations to reciprocate cooperation and punish defection are calibrated by estimates of how easily others can switch partners
Source: PLoS One. 2022 Apr 19;17(4):e0267153. doi: 10.1371/journal.pone.0267153 (PMC9017931; doi:10.1371/journal.pone.0267153)
Supplement: S1 Appendix — (DOCX) [file pone.0267153.s001.docx]

**S1 Appendix. Instructions given to participants.**

**Instructions before the Trust Game with Punishment (TGP)**

You are going to interact with other people who are participating in this study.

Other people will be your partner(s) in various situations in which you can benefit each other.

First, we will give you **points** that can be used throughout this study.

You and your partners can give each other **points** in various situations.

***Imagine* that points are something like money – e.g., the program converts points to real money at the end of the study.**

The number of points you will earn during the study *depends on both your decisions and your partners’ decisions*.

There are no right or wrong decisions. We will explain how everything works before you and your partners make any decisions.

You will be given points at the beginning of each interaction.

During that interaction, *you might lose points, depending on your decisions and your partners’ decisions*.

However, since **we will give you points for every interaction in which you engage**, you will always have a positive number of points ***in total****by the end of the study*. You will never lose money by interacting with other people in this study.

**Instructions for the TGP**

You will be either **the truster** or **the responder**.


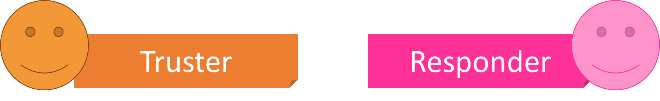


There are **four** steps.

Step 1:

**The truster** is given 100 points.


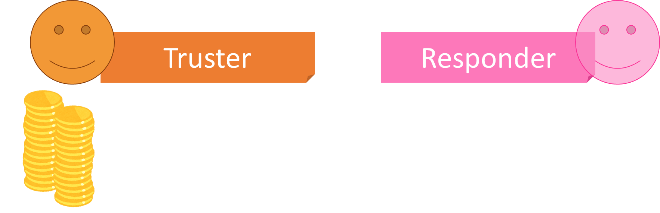


**The truster** can give **the responder** any number of these points, from 0-100.


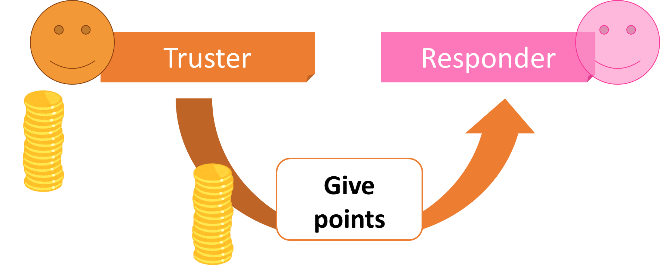


Step 2:

Whatever **the truster** gives to **the responder** is **tripled**. It becomes more valuable!


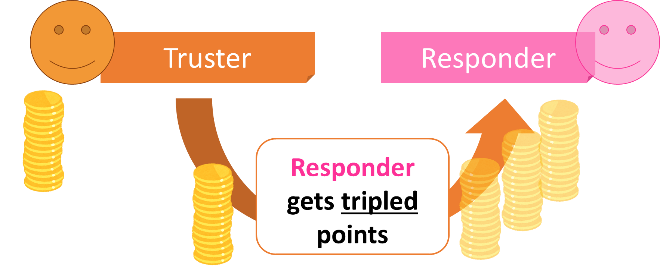


For example, suppose **the truster** gives 40 points to **the responder**.


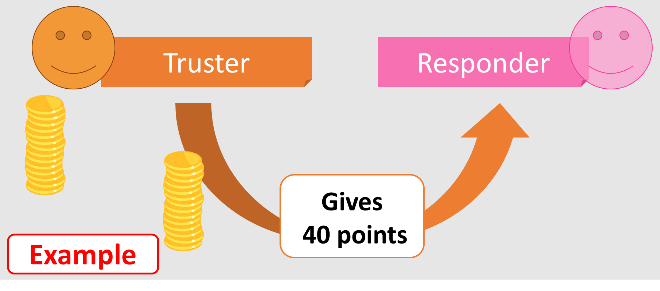


The 40 points is tripled to 120 points. So **the responder** receives 120 points, while **the truster** keeps 60 points for himself/herself.

Notice: because the points were tripled, both of you could be better off, depending on what the responder does next.


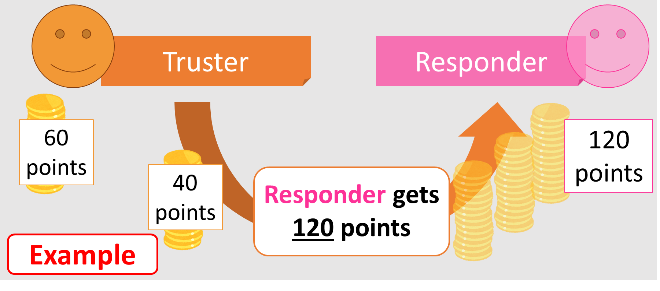


Step 3:

After receiving **the tripled points**, **the responder** can send points back to **the truster**. Any number **the responder** wants to.


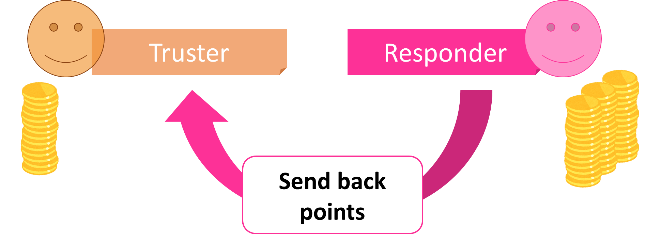


For example, if the tripled amount is 120, **the responder** can send some of those 120 points back—or none of them or all of them. As many as **the responder** wants. **The responder** keeps the points he/she did not send to **the truster**.


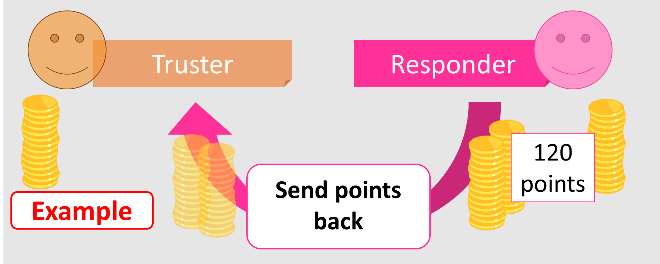


Notice: Whether **the truster** is better off than before depends **on how much of the tripled amount the responder sends back**.

For example, if the **the truster** initially gave 40 points, he/she will be better off than before if **the responder** sends back more than 40 points. But the truster will be worse off if **the responder** sends back less than 40 points.


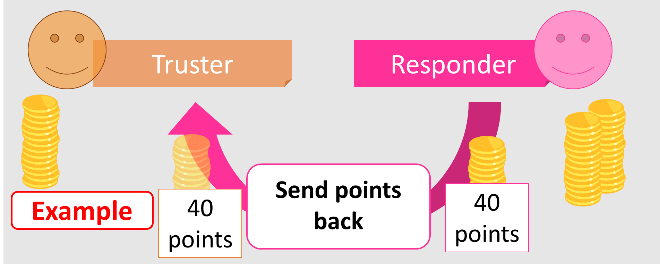


Step 4:

After receiving the points **the responder** has sent back, **the truster** can either:

(a) Do nothing (and keep all the points **the truster** now has), **OR**

**
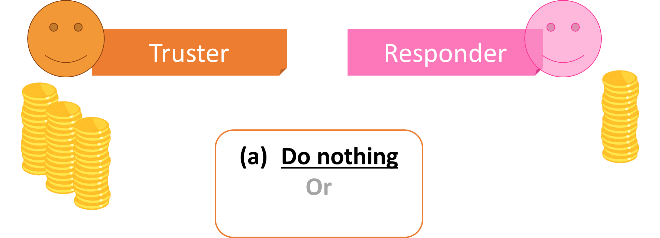
**

(b) Pay points to deduct points from **the responder**. **Every 10 points the truster** pays deducts **30 points** from **the responder**.


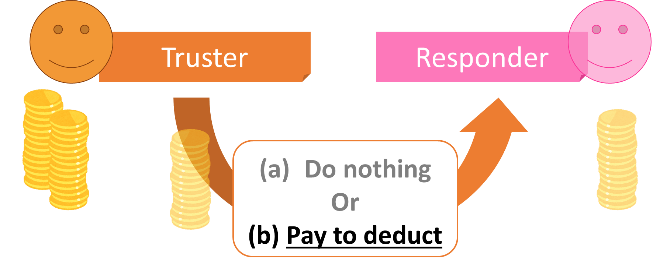


Here is a summary of the four steps.


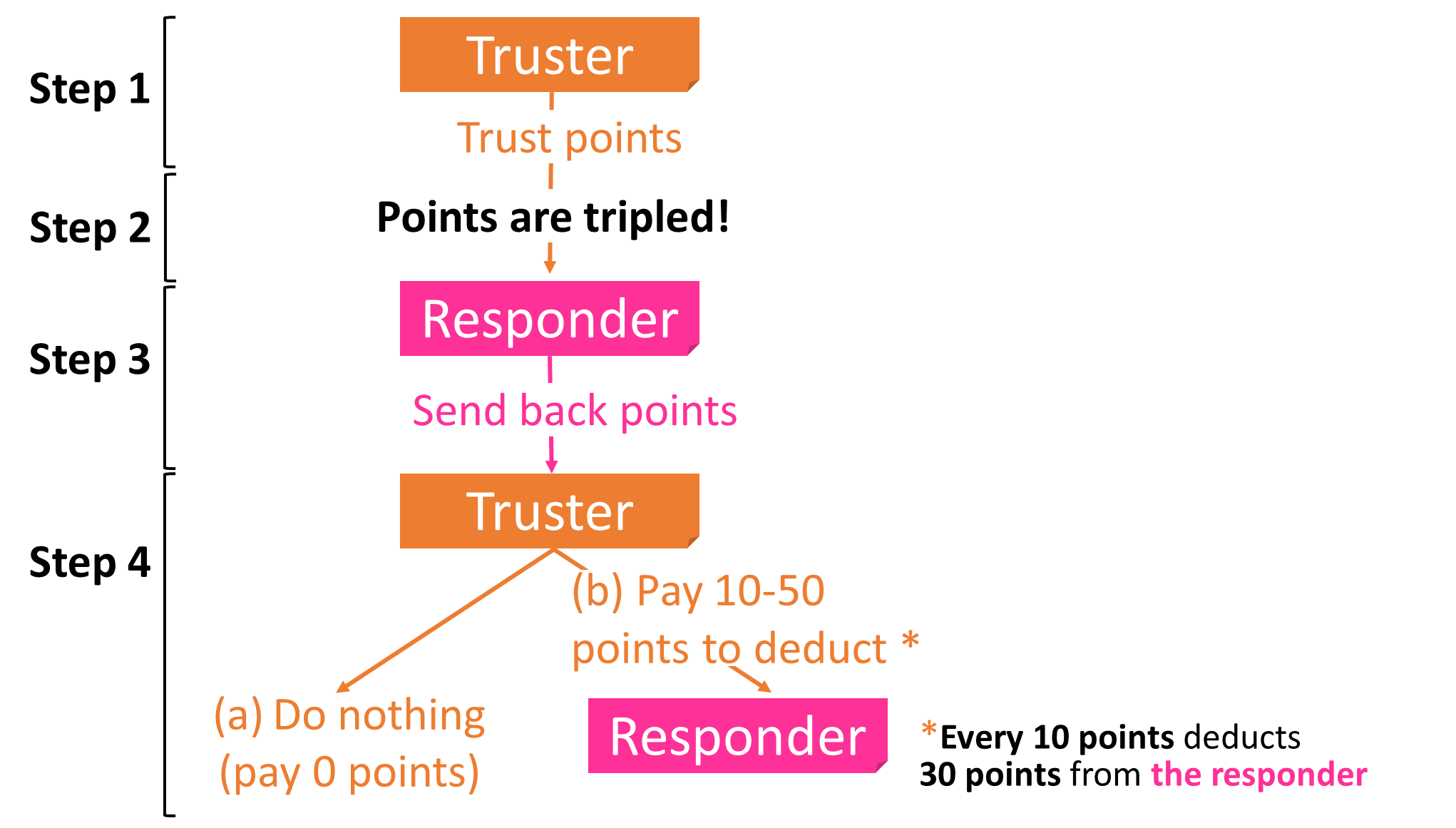


Would you like to read the explanation again?

- Yes
- No

[If Yes, go back to the top of the instruction]

**Instructions for partner switching**

We will pair you with a partner.

You will interact with the same partner **once as the responder and once as the truster with the same partner**, alternating the roles.

After interacting with the same partner **once as the responder and once as the truster (this is one block)**,

[High Partner Choice Condition]

**you will be able to** **switch partners** (if you want to).

You will keep the same partner *only if you and your partner both choose not to switch*.

[Low Partner Choice Condition]

**you will** **have the same partner for all other interactions.**

When you are paired with a partner, the program will **randomly assign you a role**, either **the truster** or **the responder**. Then you and your partner will alternate the roles.

After that, the program **will randomly decide whether you have a next block**.

**Two practice rounds of the TGP**

Let’s practice before you actually interact with your partner.

You will practice several blocks, but note that **you do not actually win or lose points during the practice session**.

After the practice session, you will be given actual points and will be able to earn more points with it.

This turn, you are **the truster** and your partner is the responder.

Now you and your partner are each given **50 points** (a bonus for you two, regardless of your role).

…

[See “Instruction during the TGP” below for details. Participants experienced two practice rounds of the TGP (once as the truster, once as the responder) and then were asked whether they wanted to switch partners in High Partner Choice condition (or they were reminded that they have the same partner in Low Partner Choice Condition). They always practiced the truster first. The sham practice partner behaved in either of the following ways (i) returns 70%, trusts 100 points, and pays 20 points to punish if being returned less than 50%, or (ii) returns 20%, trusts 30 points, and does not punish.**]**

The program has decided that **there will be no more blocks**. This ends this section of your session**.**

This concludes the practice session.

**Comprehension check questions for the TGP**

Here are a few quizzes to help you understand how you interact with your partners.

Q1.

In the **first**step, **both the truster and the responder** get 50 points (as a bonus) and **the truster gets 100 points more**.

The truster can trust the responder and give any number of points, from 0 to 100, to the responder. 

What happens to**the responder**if the truster gives **40 points** to the responder?

- The responder receives **40 points**
- The responder receives **120 points**
- The responder receives **200 points**

[If choosing the correct answer, participants proceeded to the next question. Otherwise, participants were told their answer was not correct and then given a chance to answer the same question again.]

Your answer is correct! If the truster gives **40 points** to the responder, the responder receives **the tripled points: 120 points**.

[If missing the same question twice]

Your answer is **not correct**. If the truster gives **40 points** to the responder, the responder receives **the tripled points: 120 points**.

Q2.

How many points in total would**the truster**have if he/she gave **40 points** to**the responder**?

- The truster would have 60 **points** left for himself/herself
- The truster would have **110 points left**, **50 from the initial bonus**, and **the 60 that remain after the truster gave 40 points** to the receiver.
- The truster has **40 points** left for himself/herself

[If choosing the correct answer]

Your answer is correct!

At first, the truster had 50 points (a bonus). Then, the truster was given 100 points more and asked to decide how many points they would like to give to the responder *from that 100 points*. If the truster gave 40 points to the responder, the truster would **keep 60 points out of 100 points**, but the truster **still has the original 50 points**. So the truster has 60+50 = 110.

[If missing the same question twice]

Your answer is **not correct**.

At first, the truster had 50 points (a bonus). Then, the truster was given 100 points more and asked to decide how many points they would like to give to the responder *from that 100 points*. If the truster gave 40 points to the responder, the truster would **keep 60 points out of 100 points**, but the truster **still has the original 50 points**. So the truster has 60+50 = 110.

Q3.

In the **third** step, the **responder** divides the tripled points between him/herself and **the truster**.

What happens to the truster if the responder gives **100 points**?

- The truster receives **300 points**
- The truster receives **200 points**
- The truster receives **100 points**

[If choosing the correct answer]

Your answer is correct! If the **responder** sends **100 points** back to the responder, **the truster receives what is sent by the responder**: **100 points.**

[If missing the same question twice]

Your answer is **not correct**. If the **responder** sends **100 points** back to the responder, **the truster receives what is sent by the responder**: **100 points.**

Q4.

After receiving points from the responder, the truster can either (a) do nothing (pay 0 points) or (b) **pay points to deduct points from** **the responder**.

What happens to the responder if the truster pays **10 points**?

- The responder loses **50 points**
- The responder loses **30 points**
- The responder loses **100 points**

[If choosing the correct answer]

Your answer is correct! If the truster **pays 10 points**, the responder loses **30 points. Every 10 points** the truster pays **deducts 30 points** from the responder.

[If missing the same question twice]

Your answer is **not correct**. If the truster **pays 10 points**, the responder loses **30 points. Every 10 points** the truster pays **deducts 30 points** from the responder.

Q5a. [Only for those in Low Partner Choice Condition]
After interacting with a partner for a block (both as the giver and the responder), are you going to **have the same partner**?

- Yes
- No

[If choosing the correct answer]

Your answer is correct! After the first block, **you will** **have the same partner** in the following blocks.

[If missing the same question twice]

Your answer is **not correct**. After the first block, **you will** **have the same partner** in the following blocks.

Q5b. [Only for those in High Partner Choice Condition]
After interacting with a partner for a block (both as the giver and the responder), are you going to **be able to switch to a new partner**?

- Yes
- No

[If choosing the correct answer]

Your answer is correct! After the first block, **you will be able to** **switch partners** in the following blocks. You will keep the same partner *only if you and your partner both choose not to switch*.

[If missing the same question twice]

Your answer is **not correct**. After the first block, **you will be able to** **switch partners** in the following blocks. You will keep the same partner *only if you and your partner both choose not to switch*.

**Instruction before the TGP**

Now you are going to **actually interact with your partner**.

Remember that the program **will randomly decide whether you have a next block** at the end of each block.

[High Partner Choice Condition]

Remember, after the first block, you will be **able to** **switch partners**, if you want to, for the following blocks. After each block, you will have the option to switch partners if you want to.

[Low Partner Choice Condition]

Remember, after the first block, you will **have the same partner** for all of the following blocks.

**Instruction during the TGP**

Your partner will know you as Participant **R[random two-digit number].**

Your partner is **Participant S[random two-digit number]**.

[Counter-balance: If participants became the truster]

This turn, you are **the truster** and your partner is the responder.

Now you and your partner are each given **50 points** (a bonus for you two, regardless of your role).

| *Truster* | *Responder* |
| --- | --- |
| **[Participant’s ID] (you)** | **[Sham partner’s ID] (your partner)** |
| 50 points | 50 points |

| *Truster* | *Responder* |
| --- | --- |
| **[Participant’s ID] (you)** | **[Sham partner’s ID] (your partner)** |
| 50 points | 50 points |
| [Participant’s ID], we are giving you **100 points** more.   How many points do you want to give to your partner?  Whatever you give will be **tripled.** |  |

How many points do you want to give to your partner?

- 0 points, which will become 0 points for your partner
- 10 points, which will become 30 points for your partner
- 20 points, which will become 60 points for your partner
- 30 points, which will become 90 points for your partner
- 40 points, which will become 120 points for your partner
- 50 points, which will become 150 points for your partner
- 60 points, which will become 180 points for your partner
- 70 points, which will become 210 points for your partner
- 80 points, which will become 240 points for your partner
- 90 points, which will become 270 points for your partner
- 100 points, which will become 300 points for your partner

| *Truster* | *Responder* |
| --- | --- |
| **[Participant’s ID] (you)** | **[Sham partner’s ID] (your partner)** |
| 50 points | 50 points |
| [Participant’s ID], we are giving you **100 points** more.   How many points do you want to give to your partner?  Whatever you give will be **tripled.** |  |
| [Participant’s ID], you chose to give **XX points** | Which became**3*XX points** |

| *Truster* | *Responder* |
| --- | --- |
| **[Participant’s ID] (you)** | **[Sham partner’s ID] (your partner)** |
| 50 points | 50 points |
| [Participant’s ID], we are giving you **100 points** more.   How many points do you want to give to your partner?  Whatever you give will be **tripled.** |  |
| [Participant’s ID], you chose to give **XX points** | Which became**3*XX points** |
| [Participant’s ID], you now have:  **150 - XX  points** | [Sham partner’s ID], now you have:  **3*XX + 50  points** |

| *Truster* | *Responder* |
| --- | --- |
| **[Participant’s ID] (you)** | **[Sham partner’s ID] (your partner)** |
| 50 points | 50 points |
| [Participant’s ID], we are giving you **100 points** more.   How many points do you want to give to your partner?  Whatever you give will be **tripled.** |  |
| [Participant’s ID], you chose to give **XX points** | Which became**3*XX points** |
| [Participant’s ID], you now have:  **150 - XX  points** | [Sham partner’s ID], now you have:  **3*XX + 50 points** |
|  | [Sham partner’s ID], you can now send points back to [Participant’s ID] from what he/she gave you:**3*XX points**.  How many points do you want to send back to your partner? |

| *Truster* | *Responder* |
| --- | --- |
| **[Participant’s ID] (you)** | **[Sham partner’s ID] (your partner)** |
| 50 points | 50 points |
| [Participant’s ID], we are giving you **100 points** more.   How many points do you want to give to your partner?  Whatever you give will be **tripled.** |  |
| [Participant’s ID], you chose to give **XX points** | Which became**3*XX points** |
| [Participant’s ID], you now have:  **150 - XX  points** | [Sham partner’s ID], now you have:  **3*XX + 50 points** |
|  | [Sham partner’s ID], you can now send points back to [Participant’s ID] from what he/she gave you:**3*XX points**.   How many points do you want to send back to your partner? |
| [Participant’s ID], your partner sent back **XXX points,**  **X%** of the tripled amount that you gave | [Sham partner’s ID], you sent back **XXX points**,  **X%** of what [Participant’s ID] gave to you.  You kept**XXX points**,  **X%** of it for yourself |

**[Sham partner sends back either 50% or 20%]**

| *Truster* | *Responder* |
| --- | --- |
| **[Participant’s ID] (you)** | **[Sham partner’s ID] (your partner)** |
| 50 points | 50 points |
| [Participant’s ID], we are giving you **100 points** more.   How many points do you want to give to your partner?  Whatever you give will be **tripled.** |  |
| [Participant’s ID], you chose to give **XX points** | Which became**3*XX points** |
| [Participant’s ID], you now have:  **150 - XX  points** | [Sham partner’s ID], now you have:  **3*XX + 50 points** |
|  | [Sham partner’s ID], you can now send points back to [Participant’s ID] from what he/she gave you:**3*XX points**.   How many points do you want to send back to your partner? |
| [Participant’s ID], your partner sent back **XXX points**, **X%** of the tripled amount that you gave | [Sham partner’s ID], you sent back **XXX points**,  **X%** of what [Participant’s ID] gave to you.  You kept**XXX points**,  **X%** of it for yourself |
| [Participant’s ID], you now have **XXXX points** | [Sham partner’s ID], now you have **XXXX points** |

What would you like to do?

- Do nothing (pay 0 points)
- Pay 10 points to deduct 30 points from your partner
- Pay 20 points to deduct 60 points from your partner
- Pay 30 points to deduct 90 points from your partner
- Pay 40 points to deduct 120 points from your partner
- Pay 50 points to deduct 150 points from your partner

| *Truster* | *Responder* |
| --- | --- |
| **[Participant’s ID] (you)** | **[Sham partner’s ID] (your partner)** |
| 50 points | 50 points |
| [Participant’s ID], we are giving you **100 points** more.   How many points do you want to give to your partner?  Whatever you give will be **tripled.** |  |
| [Participant’s ID], you chose to give **XX points** | Which became**3*XX points** |
| [Participant’s ID], you now have:  **150 - XX  points** | [Sham partner’s ID], now you have:  **3*XX + 50 points** |
|  | [Sham partner’s ID], you can now send points back to [Participant’s ID] from what he/she gave you:**3*XX points**.   How many points do you want to send back to your partner? |
| [Participant’s ID], your partner sent back **XXX points**, **X%** of the tripled amount that you gave | [Sham partner’s ID], you sent back **XXX points**,  **X%** of what [Participant’s ID] gave to you.  You kept**XXX points**,  **X%** of it for yourself |
| [Participant’s ID], you now have **XX points** | [Sham partner’s ID], now you have **XX points** |
| [Participant’s ID], do you want to deduct points from [Sham partner’s ID]?  **Every 10 points** you pay deduct **30 points** of [Sham partner’s ID] |  |
| [Participant’s ID], you chose to pay **XX points** to deduct **XX points**from [Sham partner’s ID] | [Sham partner’s ID], your partner deducted **XXX points** from you |

| *Truster* | *Responder* |
| --- | --- |
| **[Participant’s ID] (you)** | **[Sham partner’s ID] (your partner)** |
| 50 points | 50 points |
| [Participant’s ID], we are giving you **100 points** more.   How many points do you want to give to your partner?  Whatever you give will be **tripled.** |  |
| [Participant’s ID], you chose to give **XX points** | Which became**3*XX points** |
| [Participant’s ID], you now have:  **150 - XX  points** | [Sham partner’s ID], now you have:   **points** |
|  | [Sham partner’s ID], you can now send points back to [Participant’s ID] from what he/she gave you:**3*XX points**.   How many points do you want to send back to your partner? |
| [Participant’s ID], your partner sent back **XXX points**, **X%** of the tripled amount that you gave | [Sham partner’s ID], you sent back **XXX points**,  **X%** of what [Participant’s ID] gave to you.  You kept**XXX points**,  **X%** of it for yourself |
| [Participant’s ID], you now have **XX points** | [Sham partner’s ID], now you have **XX points** |
| [Participant’s ID], do you want to deduct points from [Sham partner’s ID]?  **Every 10 points** you pay deduct **30 points** of [Sham partner’s ID] |  |
| [Participant’s ID], you chose to pay**XX points** to deduct **XX points**from [Sham partner’s ID] | [Sham partner’s ID], your partner deducted **XXX points** from you |
| [Participant’s ID], your total is now:  **XXX points** | [Sham partner’s ID], your total is now:  **XXX points** |

-------------------------------------------------------------

Congratulations! You have earned **XXX points** as the truster.

[If participants had not played the two roles yet]

Now you and your partner alternate the roles.
**You are still paired with Participant [sham partner’s ID]**.

--------------------------------------------------------------

[Counter-balance: If the participants became the responder]

This turn, **you are the responder** and your partner is the truster.

Now you and your partner are each given **50 points** (a bonus for you two, regardless of your role).

| *Responder* | *Truster* |
| --- | --- |
| **[Participant’s ID] (you)** | **[Sham partner’s ID] (your partner)** |
| 50 points | 50 points |

| *Responder* | *Truster* |
| --- | --- |
| **[Participant’s ID] (you)** | **[Sham partner’s ID] (your partner)** |
| 50 points | 50 points |
|  | [Sham partner’s ID], we are giving you **100 points**more.   How many points do you want to give to your partner?  Whatever you give will be **tripled**. |

| *Responder* | *Truster* |
| --- | --- |
| **[Participant’s ID] (you)** | **[Sham partner’s ID] (your partner)** |
| 50 points | 50 points |
|  | [Sham partner’s ID], we are giving you **100 points**more.   How many points do you want to give to your partner?  Whatever you give will be **tripled**. |
| Which became **210 points** | [Sham partner’s ID], you chose to give **70 points** |

| *Responder* | *Truster* |
| --- | --- |
| **[Participant’s ID] (you)** | **[Sham partner’s ID] (your partner)** |
| 50 points | 50 points |
|  | [Sham partner’s ID], we are giving you **100 points**more.   How many points do you want to give to your partner?  Whatever you give will be **tripled**. |
| Which became **210 points** | [Sham partner’s ID], you chose to give **70 points** |
| [Participant’s ID], you now have:  **260 points** | [Sham partner’s ID], now you have:  **130 points** |

| *Responder* | *Truster* |
| --- | --- |
| **[Participant’s ID] (you)** | **[Sham partner’s ID] (your partner)** |
| 50 points | 50 points |
|  | [Sham partner’s ID], we are giving you **100 points**more.   How many points do you want to give to your partner?  Whatever you give will be **tripled**. |
| Which became **210 points** | [Sham partner’s ID], you chose to give **70 points** |
| [Participant’s ID], you now have:  **260 points** | [Sham partner’s ID], now you have:  **130 points** |
| [Participant’s ID], you can now send points back to [Sham partner’s ID] from what he/she gave you: XXX points.  How many points do you want to send back to your partner? |  |

How many points do you want to send back to your partner?

- XX points, 0% of what your partner gave you
- XX points, 10% of what your partner gave you
- XX points, 20% of what your partner gave you
- XX points, 30% of what your partner gave you
- XX points, 40% of what your partner gave you
- XX points, 50% of what your partner gave you
- XX points, 60% of what your partner gave you
- XX points, 70% of what your partner gave you
- XX points, 80% of what your partner gave you
- XX points, 90% of what your partner gave you
- XX points, 100% of what your partner gave you

| *Responder* | *Truster* |
| --- | --- |
| **[Participant’s ID] (you)** | **[Sham partner’s ID] (your partner)** |
| 50 points | 50 points |
|  | [Sham partner’s ID], we are giving you **100 points**more.   How many points do you want to give to your partner?  Whatever you give will be **tripled**. |
| Which became **210 points** | [Sham partner’s ID], you chose to give **70 points** |
| [Participant’s ID], you now have:  **260 points** | [Sham partner’s ID], now you have:  **130 points** |
| [Participant’s ID], you can now send points back to [Sham partner’s ID] from what he/she gave you: XXX points.  How many points do you want to send back to your partner? |  |
| [Participant’s ID], you sent back **XXX points**,  **X%** of what [Sham partner’s ID] gave to you.  You kept**XX points**,  **X%** of it for yourself | [Sham partner’s ID], your partner sent back **XXX points**,  **X%** of the tripled amount that you gave |

| *Responder* | *Truster* |
| --- | --- |
| **[Participant’s ID] (you)** | **[Sham partner’s ID] (your partner)** |
| 50 points | 50 points |
|  | [Sham partner’s ID], we are giving you **100 points**more.   How many points do you want to give to your partner?  Whatever you give will be **tripled**. |
| Which became **210 points** | [Sham partner’s ID], you chose to give **70 points** |
| [Participant’s ID], you now have:  **260 points** | [Sham partner’s ID], now you have:  **130 points** |
| [Participant’s ID], you can now send points back to [Sham partner’s ID] from what he/she gave you: XXX points.  How many points do you want to send back to your partner? |  |
| [Participant’s ID], you sent back **XXX points**,  **X%** of what [Sham partner’s ID] gave to you.  You kept**XX points**,  **X%** of it for yourself | [Sham partner’s ID], your partner sent back **XXX points**,  **X​%** of the tripled amount that you gave |
| [Participant’s ID], you now have **XXX points** | [Sham partner’s ID], now you have**XXX points** |

|  |  |
| --- | --- |
| *Responder* | *Truster* |
| **[Participant’s ID] (you)** | **[Sham partner’s ID] (your partner)** |
| 50 points | 50 points |
|  | [Sham partner’s ID], we are giving you **100 points**more.   How many points do you want to give to your partner?  Whatever you give will be **tripled**. |
| Which became **210 points** | [Sham partner’s ID], you chose to give **70 points** |
| [Participant’s ID], you now have:  **260 points** | [Sham partner’s ID], now you have:  **130 points** |
| [Participant’s ID], you can now send points back to [Sham partner’s ID] from what he/she gave you: XXX points.  How many points do you want to send back to your partner? |  |
| [Participant’s ID], you sent back **XXX points**,  **X%** of what [Sham partner’s ID] gave to you.  You kept**XX points**,  **X%** of it for yourself | [Sham partner’s ID], your partner sent back **XXX points**,  **X​%** of the tripled amount that you gave |
| [Participant’s ID], you now have **XXX points** | [Sham partner’s ID], now you have**XXX points** |
|  | [Sham partner’s ID], do you want to deduct points from [Participant’s ID]  **Every 10 points** you pay deduct **30 points** of [Participant’s ID] |

| *Responder* | *Truster* |
| --- | --- |
| **[Participant’s ID] (you)** | **[Sham partner’s ID] (your partner)** |
| 50 points | 50 points |
|  | [Sham partner’s ID], we are giving you **100 points**more.   How many points do you want to give to your partner?  Whatever you give will be **tripled**. |
| Which became **210 points** | [Sham partner’s ID], you chose to give **70 points** |
| [Participant’s ID], you now have:  **260 points** | [Sham partner’s ID], now you have:  **130 points** |
| [Participant’s ID], you can now send points back to [Sham partner’s ID] from what he/she gave you: XXX points.  How many points do you want to send back to your partner? |  |
| [Participant’s ID], you sent back **XXX points**,  **X%** of what [Sham partner’s ID] gave to you.  You kept**XX points**,  **X%** of it for yourself | [Sham partner’s ID], your partner sent back **XXX points**,  **X​%** of the tripled amount that you gave |
| [Participant’s ID], you now have **XXX points** | [Sham partner’s ID], now you have**XXX points** |
|  | [Sham partner’s ID], do you want to deduct points from [Participant’s ID]  **Every 10 points** you pay deduct **30 points** of [Participant’s ID] |
| [Participant’s ID], your partner deducted  **XXX points**  from you | [Sham partner’s ID], you chose to pay **XXX points** to **deduct XXX points** from [Participant’s ID] |

[If participants return less than 50%, there was a 50% chance that the sham truster pays 20 points to deduct 60 points from the participant]

| *Responder* | *Truster* |
| --- | --- |
| **[Participant’s ID] (you)** | **[Sham partner’s ID] (your partner)** |
| 50 points | 50 points |
|  | [Sham partner’s ID], we are giving you **100 points**more.   How many points do you want to give to your partner?  Whatever you give will be **tripled**. |
| Which became **210 points** | [Sham partner’s ID], you chose to give **70 points** |
| [Participant’s ID], you now have:  **260 points** | [Sham partner’s ID], now you have:  **130 points** |
| [Participant’s ID], you can now send points back to [Sham partner’s ID] from what he/she gave you: XXX points.  How many points do you want to send back to your partner? |  |
| [Participant’s ID], you sent back **XXX points**,  **X%** of what [Sham partner’s ID] gave to you.  You kept**XX points**,  **X%** of it for yourself | [Sham partner’s ID], your partner sent back **XXX points**,  **X​%** of the tripled amount that you gave |
| [Participant’s ID], you now have **XXX points** | [Sham partner’s ID], now you have**XXX points** |
|  | [Sham partner’s ID], do you want to deduct points from [Participant’s ID]  **Every 10 points** you pay deduct **30 points** of [Participant’s ID] |
| [Participant’s ID], your partner deducted  **XXX points**  from you | [Sham partner’s ID], you chose to pay **XXX points** to **deduct XXX points** from [Participant’s ID] |
| [Participant’s ID], your total is now:  **XXX points** | [Sham partner’s ID], your total is now:  **XXX points** |

Congratulations! You have earned **XXX points** as the responder.

-------------------------------------------------------------

[If participants had played the two roles]

This concludes the first block.

**Partner switching after the TGP**

[High Partner Choice Condition]

Now you are able to switch partners. **You can either keep Participant [Sham partner’s ID] or switch to a different partner**.

Would you like to switch partners?

I would like to:

- Keep the same partner
- Switch to a different partner

[If choosing “Keep the same partner”]

You will continue to interact with **your former partner, Participant [Sham partner’s ID] in the next block.**

[If choosing “Switch to a different partner”]

You now have **a different partner, Participant [random two-digit number].**

[Low Partner Choice Condition]

You will continue to interact with **your former partner, Participant [Sham partner’s ID].**

**After partner switching**

**The program has decided that there will be no more blocks**. This ends this section of your session**.**

**This concludes your interaction with other people.**
