# Supplementary material for: Motivations to reciprocate cooperation and punish defection are calibrated by estimates of how easily others can switch partners
Source: PLoS One. 2022 Apr 19;17(4):e0267153. doi: 10.1371/journal.pone.0267153 (PMC9017931; doi:10.1371/journal.pone.0267153)
Supplement: S3 Appendix — (DOCX) [file pone.0267153.s003.docx]

**S3 Appendix. Full regression models.**

**Partner switching**

Table S1 presents the effects of predictors of the decision to switch partners (DV3. See the main text Section 3.1 for descriptions of predictors). In every analysis reported here, continuous variables were centered by subtracting the mean as described in the main text Section 3.3.

| **S1 Table. Factors affecting the decision to switch partners.** | | | | | | |  |
| --- | --- | --- | --- | --- | --- | --- | --- |
| Predictors | *b* | *SE* | Wald χ2 | *OR* | 95% CI | *p* | |
| i. Reciprocation by the participant (0-100) | 0.0009 | 0.01 | 0.02 | 1.00 | [0.99, 1.01] | .883 | |
| ii. Punishment received (1, 0) | 1.13 | 0.28 | 16.36 | 3.10 | [1.80, 5.40] | < .001 | |
| iii. Trust (0-100) | 0.01 | 0.00 | 11.02 | 1.01 | [1.00, 1.02] | .001 | |
| iv. Defection by the responder (1, 0) | 1.49 | 0.22 | 46.13 | 4.45 | [2.91, 6.89] | < .001 | |
| v. Amount paid to punish the responder (0-50) | 0.02 | 0.01 | 7.46 | 1.02 | [1.01, 1.03] | .006 | |
| Society: US (vs. Japan) | -0.45 | 0.21 | 4.45 | 0.64 | [0.42, 0.97] | .035 | |
| Note. Nagelkerke pseudo R^2^ = 0.26. CI = confidence interval for *OR*. VIF values were < 1.6. | | | | | | |  |

**Punishment**

Table S2 presents the effects of predictors on *Amount paid to punish the responder* (DV2), controlling for whether the responder defected or not. Table S3 presents effects of predictors when the responder defected (returned 20% of what they received from participants). See the main text Section 3.2 for descriptions of predictors.

| **S2 Table. Factors affecting the amount paid to punish the responder.** | | | | | |  |
| --- | --- | --- | --- | --- | --- | --- |
| Predictors | *b* | *SE* | 95% CI | *β* | *t* | *p* |
| Condition: High Partner Choice (vs. Low) | -0.06 | 1.18 | [-2.39, 2.27] | -0.002 | -0.05 | .961 |
| Society: US (vs. Japan) | 3.82 | 1.30 | [1.27, 6.37] | 0.13 | 2.94 | .003 |
| *RM others* | -2.89 | 0.92 | [-4.71, -1.08] | -0.14 | -3.13 | .002 |
| Defection by the responder (1, 0) | 8.49 | 1.19 | [6.15, 10.82] | 0.30 | 7.15 | < .001 |
| Trust (0-100) | 0.06 | 0.02 | [0.02, 0.10] | 0.14 | 3.34 | < .001 |
| Note. Adjusted R^2^ = 0.12. CI = confidence interval for *b*. VIF values were < 1.3. | | | | |  |  |

| **S3 Table. Factors affecting the amount paid to punish the responder who had defected.** | | | | | | |
| --- | --- | --- | --- | --- | --- | --- |
| Predictors | *b* | *SE* | 95% CI | *β* | *t* | *p* |
| Condition: High Partner Choice (vs. Low) | -0.86 | 2.07 | [-4.94, 3.23] | -0.03 | -0.41 | .679 |
| Society: US (vs. Japan) | 3.65 | 2.25 | [-0.79, 8.09] | 0.11 | 1.62 | .107 |
| *RM others* | -4.79 | 1.63 | [-8.00, -1.59] | -0.20 | -2.95 | .004 |
| Trust (0-100) | 0.16 | 0.03 | [0.10, 0.22] | 0.31 | 5.00 | < .001 |
| Note. Adjusted R^2^ = 0.10. CI = confidence interval for *b*. VIF values were < 1.3. | | | | |  |  |

**Reciprocation**

Table S4 presents the effects of predictors on *Reciprocation by the participant* (DV1). As we found interactions that involve society (US versus Japan), we separately conducted the same regression for each society. Table S5 represents the effects of predictors on reciprocation only by Japanese participants. Table S6 represents the effects of predictors on reciprocation by American participants. See main text Section 3.3 for descriptions of predictors.

| **S4 Table. Factors affecting reciprocation by the participant.** | | | | | |  |
| --- | --- | --- | --- | --- | --- | --- |
| Predictors | *b* | *SE* | 95% CI | *β* | *t* | *p* |
| Condition: High Partner Choice (vs. Low) | 4.43 | 2.82 | [-1.12, 9.97] | 0.10 | 1.57 | .118 |
| Society: US (vs. Japan) | -5.68 | 2.71 | [-10.99, -0.36] | -0.13 | -2.10 | .036 |
| *RM others* | 8.38 | 2.49 | [3.49, 13.27] | 0.27 | 3.37 | < .001 |
| Condition × Society | -9.00 | 3.96 | [-16.78, -1.22] | -0.18 | -2.27 | .023 |
| Condition × *RM others* | -5.74 | 3.87 | [-13.35, 1.87] | -0.13 | -1.48 | .139 |
| Society × *RM others* | -11.41 | 3.93 | [-19.14, -3.69] | -0.24 | -2.90 | .004 |
| Condition × Society × *RM others* | 15.80 | 5.70 | [4.61, 26.99] | 0.24 | 2.77 | .006 |
| Note. Adjusted R^2^ = 0.07. CI = confidence interval for *b*. VIF values were < 4.4. | | | | | | |

| **S5 Table. Factors affecting reciprocation by Japanese participants.** | | | | | |  |
| --- | --- | --- | --- | --- | --- | --- |
| Predictors | *b* | *SE* | 95% CI | *β* | *t* | *p* |
| Condition: High Partner Choice (vs. Low) | 4.43 | 3.07 | [-1.63, 10.48] | 0.10 | 1.44 | .151 |
| *RM others* | 8.38 | 2.71 | [3.04, 13.72] | 0.25 | 3.09 | .002 |
| Condition × *RM others* | -5.74 | 4.22 | [-14.05, 2.56] | -0.11 | -1.36 | .175 |
| Note. Adjusted R^2^ = 0.04. CI = confidence interval for *b*. VIF values were < 2.0. | | | | | | |

| **S6 Table. Factors affecting reciprocation by American participants.** | | | | | |  |
| --- | --- | --- | --- | --- | --- | --- |
| Predictors | *b* | *SE* | 95% CI | *β* | *t* | *p* |
| Condition: High Partner Choice (vs. Low) | -4.57 | 2.52 | [-9.53, 0.38] | -0.12 | -1.82 | .070 |
| *RM others* | -3.03 | 2.76 | [-8.46, 2.40] | -0.10 | -1.10 | .273 |
| Condition × *RM others* | 10.05 | 3.78 | [2.60, 17.51] | 0.24 | 2.66 | .008 |
| Note. Adjusted R^2^ = 0.02. CI = confidence interval for *b*. VIF values were < 2.3. | | | | | | |
