# Supplementary material for: Motivations to reciprocate cooperation and punish defection are calibrated by estimates of how easily others can switch partners
Source: PLoS One. 2022 Apr 19;17(4):e0267153. doi: 10.1371/journal.pone.0267153 (PMC9017931; doi:10.1371/journal.pone.0267153)
Supplement: S4 Appendix — (DOCX) [file pone.0267153.s004.docx]

**S4 Appendix. Full logistic regression models for the decision to switch partners given different reciprocation rates by the participant.**

Table S7 shows the effects of predictors on the decision to switch partners in participants who could be punished: that is, those who returned 40% or less (*n* = 227).

Table S8-1 shows the same analysis for those who returned exactly 40% (*n* = 79). These participants delivered a positive payoff to their partner, but had a higher payoff than their partner.

Table S8-2 shows the analysis for defectors: participants who inflicted a negative payoff on their partner by returning 30% or less (*n* = 148). See the main text Section 3.1 for descriptions of predictors.

| **S7 Table. Factors affecting the decision to switch partners for those who returned 40% or less.** | | | | | | | |
| --- | --- | --- | --- | --- | --- | --- | --- |
| Predictors | *b* | *SE* | Wald χ2 | *OR* | 95% CI | *p* |  |
| i. Reciprocation by the participant (0-40) | 0.007 | 0.01 | 0.36 | 1.01 | [0.98, 1.03] | .548 |  |
| ii. Punishment received (1, 0) | 1.21 | 0.31 | 15.05 | 3.35 | [1.84, 6.27] | < .001 |  |
| iii. Trust (0-100) | 0.01 | 0.00 | 7.13 | 1.01 | [1.00, 1.02] | .008 |  |
| iv. Defection by the responder (1, 0) | 1.28 | 0.32 | 15.69 | 3.60 | [1.93, 6.89] | < .001 |  |
| v. Amount paid to punish the responder (0-50) | 0.01 | 0.01 | 1.51 | 1.01 | [0.99, 1.03] | .219 |  |
| Society: US (vs. Japan) | -0.02 | 0.31 | 0.003 | 0.98 | [0.54, 1.80] | .956 |  |
| Note. Nagelkerke pseudo R^2^ = 0.26. CI = confidence interval for *OR*. VIF values were < 1.13. | | | | | | | |

| **S8-1 Table. Factors affecting the decision to switch partners for those who returned exactly 40%.** | | | | | | |
| --- | --- | --- | --- | --- | --- | --- |
| Predictors | *b* | *SE* | Wald χ2 | *OR* | 95% CI | *p* |
| i. Reciprocation by the participant (40) | N/A | N/A | N/A | N/A | N/A | N/A |
| ii. Punishment received (1, 0) | 2.26 | 0.67 | 11.43 | 9.58 | [2.82, 40.81] | < .001 |
| iii. Trust (0-100) | -0.003 | 0.01 | 0.11 | 1.00 | [0.98, 1.02] | .744 |
| iv. Defection by the responder (1, 0) | 1.39 | 0.70 | 3.95 | 4.01 | [1.01, 17.54] | .047 |
| v. Amount paid to punish the responder (0-50) | 0.05 | 0.02 | 5.21 | 1.05 | [1.01, 1.11] | .023 |
| Society: US (vs. Japan) | -1.36 | 0.63 | 4.62 | 0.26 | [0.07, 0.85] | .032 |
| Note. Nagelkerke pseudo R^2^ = 0.497. CI = confidence interval for *OR*. VIF values were < 1.42. | | | | | | |

| **S8-2 Table. Factors affecting the decision to switch partners for those who returned 30% or less.** | | | | | | |
| --- | --- | --- | --- | --- | --- | --- |
| Predictors | *b* | *SE* | Wald χ2 | *OR* | 95% CI | *p* |
| i. Reciprocation by the participant (0-30) | -0.02 | 0.02 | 1.06 | 0.98 | [0.95, 1.02] | .303 |
| ii. Punishment received (1, 0) | 0.69 | 0.39 | 3.15 | 1.99 | [0.94, 4.33] | .076 |
| iii. Trust (0-100) | 0.02 | 0.01 | 12.06 | 1.02 | [0.17, 0.87] | < .001 |
| iv. Defection by the responder (1, 0) | 1.13 | 0.41 | 7.78 | 3.11 | [1.43, 7.10] | .005 |
| v. Amount paid to punish the responder (0-50) | 0.005 | 0.01 | 0.13 | 1.00 | [0.98, 1.03] | .720 |
| Society: US (vs. Japan) | 0.71 | 0.40 | 3.10 | 2.03 | [0.94, 4.59] | .078 |
| Note. Nagelkerke pseudo R^2^ = 0.256. CI = confidence interval for *OR*. VIF values were < 1.11. | | | | | | |
