# Supplementary material for: Motivations to reciprocate cooperation and punish defection are calibrated by estimates of how easily others can switch partners
Source: PLoS One. 2022 Apr 19;17(4):e0267153. doi: 10.1371/journal.pone.0267153 (PMC9017931; doi:10.1371/journal.pone.0267153)
Supplement: S5 Appendix — (DOCX) [file pone.0267153.s005.docx]

**S5 Appendix. Regression models for behaviors in round 2.**

We conducted additional multiple linear regression models to determine what predicts participants behavior in round 2 of the TGP. The main text shows regressions for their behavior in round 1, because we wanted to examine the effects of predictors *before* they learned how the current partner treated them personally. Here, we examine whether the behaviors in round 1 affect how participants treated the partner in round 2.

**S5.1 Punishment in round 2**

First, we examine what predicts the *Amount paid to punish the partner* in round 2 (0-50 points). We only analyzed those who played the truster role in round 2 and were then defected on by their partner (*n* = 258).

We controlled for the same variables in the regression that analyzed round 1 (see the main text Section 3.1). That is, we controlled for condition (High Partner Choice = 1, Low Partner Choice = 0), society (US = 1, Japan = 0), *RM others*, and how many points the participant sent to their partner in round 2 (*Trust* in round 2: 0-100 points).

In this analysis, we added two predictors from round 1: (i) whether the sham truster punished the participant in round 1 (*Punishment received*: 1 = punished, 0 = not punished) and (ii) how many points participants returned in round 1 (*Reciprocation by the participant*: 0-40%).

In addition, for all analyses reported in this Appendix, we entered interactions between the predictors and used stepwise selection in R [54] to determine the best model by AIC. After centering the continuous variables (see the main text Section 3.1), no evidence of multicollinearity was observed in any of the analyses reported here.

Table S9-1 presents the effects of predictors on the amount paid to punish the responder who had defected in round 2. When the behaviors in round 1 were controlled for, relational mobility did not influence participants’ motivation to punish the defecting partner (*RM others*: β = -.11, *p* = .092). Additionally, being punished by the partner in round 1 increased the amount participants paid to punish the partner in round 2 (*Punishment received* in round 1: β = .19, *p* = .007). Directions of other predictors are in line with the analyses for punishment in round 1 (see S2 Table in S3 Appendix).

| **S9-1 Table. Factors affecting the amount paid to punish those who defected in round 2.** | | | | | | | | | |
| --- | --- | --- | --- | --- | --- | --- | --- | --- | --- |
| Predictors | *b* | *SE* | 95% CI | | *β* | | *t* | | *p* |
| Condition: High Partner Choice (vs. Low) | -4.08 | 3.50 | [-10.97, 2.81] | | -0.10 | | -1.17 | | .245 |
| Society: US (vs. Japan) | 3.64 | 3.35 | [-2.95, 10.23] | | 0.09 | | 1.09 | | .278 |
| *RM others* | -3.15 | 1.86 | [-6.82, 0.52] | | -0.11 | | -1.69 | | .092 |
| Trust (0-100) in round 2 | 0.15 | 0.05 | [0.05, 0.25] | | 0.25 | | 2.97 | | .003 |
| Punishment received (0, 1) in round 1 | 8.68 | 3.16 | [2.45, 14.91] | | 0.19 | | 2.74 | | .007 |
| Reciprocation by the participant (0-100) in round 1 | 0.13 | 0.07 | [-0.01, 0.26] | | 0.14 | | 1.88 | | .061 |
| Condition x Society | 7.45 | 4.76 | [-1.93, 16.82] | | 0.17 | | 1.56 | | .119 |
| Trust in round 2 x Punishment received in round 1 | 0.13 | 0.08 | [-0.03, 0.30] | | 0.12 | | 1.56 | | .121 |
| Note. Adjusted R^2^ = 0.13. CI = confidence interval for *b*. VIF values were < 3.29. | | | |  | |  | |  | |

The above analysis indicates that people engaged in retaliatory punishment—they paid more points to punish a partner who had punished them in round 1.

Next we examine whether being punished by the partner in round 1 made participants more punitive in round 2, regardless of whether the partner had reciprocated or defected in round 2. This broadens the analysis to include anti-social punishment in round 2 (along with punishment to defectors). To do this, we added another predictor: whether the partner reciprocated or defected in round 2 (*Defection by the responder* in round 2: 0 = reciprocated; 1 = defected). We limited the analysis to those who *could* have been punished in round 1—that is, those who returned 40% or less in round 1 (*n* = 236). 50% of these individuals were punished.

Table S9-2 presents the effects of predictors on retaliatory punishment. Having been punished by the partner in round 1 significantly increased the amount of punishment they inflicted on the same partner in round 2 (β = .23, *p* = .0002) when controlling for other factors. That is, people engaged in retaliatory punishment: People paid more points to punish the partners who had punished them than the partners who had not. Notice that retaliatory punishment was observed regardless of whether the responder defected. Controlling for the effect of *Defection by the responder* (β = .23, *p* = .0001), punishment received in round 1 still significantly increased punishment in round 2.

| **S9-2 Table. Factors affecting the amount paid to punish in round 2 for those who returned 40% or less in round 1 .** | | | | | | | | |
| --- | --- | --- | --- | --- | --- | --- | --- | --- |
| Predictors | *b* | *SE* | 95% CI | *β* | | *t* | | *p* |
| *RM others* | -2.68 | 1.34 | [-5.32, -0.04] | -0.12 | | -2.00 | | .047 |
| Trust (0-100) in round 2 | 0.08 | 0.04 | [0.002, 0.16] | 0.17 | | 2.03 | | .043 |
| Defection by the responder (1, 0) in round 2 | 7.21 | 1.85 | [3.56, 10.86] | 0.23 | | 3.89 | | < .001 |
| Punishment received (0, 1) in round 1 | 7.20 | 1.89 | [3.48, 10.92] | 0.23 | | 3.81 | | < .001 |
| Trust in round 2 x Punishment received in round 1 | 0.12 | 0.06 | [0.01, 0.23] | 0.18 | | 2.17 | | .031 |
| Note. Adjusted R^2^ = 0.18. CI = confidence interval for *b*. VIF values were < 2.08. | | | |  |  | |  | |

Whereas relational mobility did not predict punishment of defectors in round 2 (Table S9-1), it did predict round 2 punishment in this analysis, which includes anti-social punishment (*RM others*: β = -.12, *p* = .047).

**S5.1.1. Punishment in round 2 in the US and Japan**

Although we did not find effects of society on punishment in round 2, we separately analyzed American (*n* = 133) and Japanese (*n* = 103) participants. Note that we again limited analyses to those who could have been punished in round 1.

In the US, there was a three-way interaction involving *Reciprocation by the participant* in round 1, *Punishment received* in round 1, and *Defection by the responder* in round 2 (β = -0.32, *p* =.035). So we analyzed those who experienced defection in round 2 (*n* = 72) separately from those who did not (*n* = 61).

When the partner defected in round 2, we replicated the pattern when the two societies were analyzed together (see Table S9-3a). Participants paid more to punish when they trusted more (*Trust*: β = 0.48, *p* =.10^-5^) and when they had been punished in round 1 (*Punishment received*: β = 0.24, *p* =.028). These are the only significant predictors that remained in the model.

| **S9-3a Table. Factors affecting the amount Americans paid to punish the defecting responder in round 2 (those returning 40% or less in round 1).** | | | | | | | | |
| --- | --- | --- | --- | --- | --- | --- | --- | --- |
| Predictors | *b* | *SE* | 95% CI | *β* | | *t* | | *p* |
| Condition: High Partner Choice (vs. Low) | 1.88 | 4.09 | [-6.29, 10.06] | 0.05 | | 0.46 | | .647 |
| *RM others* | 3.21 | 6.02 | [-8.82, 15.24] | 0.10 | | 0.53 | | .596 |
| Trust (0-100) in round 2 | 0.25 | 0.06 | [0.14, 0.36] | 0.48 | | 4.44 | | < .001 |
| Punishment received (0, 1) in round 1 | 9.09 | 4.06 | [1.00, 17.19] | 0.24 | | 2.24 | | .028 |
| Condition x *RM others* | -10.96 | 7.21 | [-25.35, 3.43] | -0.29 | | -1.52 | | .133 |
| Note. Adjusted R^2^ = 0.22. CI = confidence interval for *b*. VIF values were < 3.54. | | | | |  | |  | |

Results were different, however, for participants who experienced reciprocation in round 2. The effects of *Trust* in round 2 and *Punishment received* in round 1 were insignificant and therefore removed from the model (see Table S9-3b). For these participants, condition (β = -.28, *p* = .025) and *RM others* (β = -.31, *p* = .012) remained in the model, and both decreased the *amount paid to punish the responder*.

| **S9-3b Table. Factors affecting the amount Americans paid to punish the reciprocating responder in round 2 (those returning 40% or less in round 1).** | | | | | | |  |
| --- | --- | --- | --- | --- | --- | --- | --- |
| Predictors | *b* | *SE* | 95% CI | *β* | *t* | *p* | |
| Condition: High Partner Choice (vs. Low) | -6.04 | 2.62 | [-11.27, -0.80] | -0.28 | -2.31 | .025 | |
| *RM others* | -5.47 | 2.12 | [-9.70, -1.23] | -0.31 | -2.59 | .012 | |
| Note. Adjusted R^2^ = 0.13. CI = confidence interval for *b*. VIF values were < 1.01. | | | | |  |  | |

In Japanese participants, we replicated the pattern when the two societies were analyzed together (see Table S9-4). Regardless of whether the partner defected or not, those who experienced punishment in round 1 paid more to punish the partner in round 2 (β = .29, *p* = .001), and this tendency to retaliate was escalated when the participant risked more points in round 2 (interaction between *Trust* in round 2 and *Punishment received* in round 1: β = .36, *p* = .006). Neither *RM others* nor condition remained in the model.

| **S9-4 Table. Factors affecting the amount Japanese participants paid to punish the responder in round 2 (those returning 40% or less in round 1).** | | | | | | | | | |
| --- | --- | --- | --- | --- | --- | --- | --- | --- | --- |
| Predictors | *b* | *SE* | 95% CI | | *β* | | *t* | | *p* |
| Trust (0-100) in round 2 | 0.04 | 0.07 | [-0.10, 0.17] | | 0.07 | | 0.56 | | .576 |
| Reciprocation by the participant (0-40) in round 1 | -4.42 | 2.57 | [-9.51, 0.67] | | -0.15 | | -1.72 | | .088 |
| Punishment received (0, 1) in round 1 | 8.65 | 2.61 | [3.48, 13.82] | | 0.29 | | 3.32 | | .001 |
| Trust in round 2 x Punishment received in round 1 | 0.26 | 0.09 | [0.08, 045] | | 0.36 | | 2.79 | | .006 |
| Note. Adjusted R^2^ = 0.23. CI = confidence interval for *b*. VIF values were < 2.25. | | | |  | |  | |  | |

**S5.2 Reciprocation in round 2**

Now we examine what predicts *reciprocation by the participant* in round 2 (the percent of 3P points that the participant returned to the truster: 0-100%) (*n* = 509). We controlled for the same variables in the regression analyzing round 1 (see the main text Section 3.3): condition (High Partner Choice = 1, Low Partner Choice = 0), society (US = 1, Japan = 0), and *RM other*s. We added three new predictors from round 1:

(i) how many points the participant sent to the partner in round 1 (*Trust*: 0-100)

(ii) whether the partner defected or reciprocated in round 1 (*Defection by the responder*: 0 = reciprocated; 1 = defected)

(iii) the points participants paid to punish the partner in round 1 (*Amount paid to punish the responder*: 0-50).

Table S10-1 presents the effects of predictors on *Reciprocation* *by the participant* in round 2. What participants experienced in round 1 affected how much they reciprocated in round 2. First, participants reciprocated less in round 2 when the partner had defected on them in round 1 than when the partner had reciprocated (*Defection by the responder* in round 1: β = -.23, *p* = 10^-7^). Second, those who had sent more points to the partner in round 1 reciprocated more in round 2 (*Trust* in round 1: β = .15, *p* = .008).

| **S10-1 Table. Factors affecting reciprocation by the participant in round 2.** | | | |  |  |  |
| --- | --- | --- | --- | --- | --- | --- |
| Predictors | *b* | *SE* | 95% CI | *β* | *t* | *p* |
| Condition: High Partner Choice (vs. Low) | -3.35 | 2.41 | [-8.09, 1.39] | -0.08 | -1.39 | .165 |
| Society: US (vs. Japan) | -12.61 | 2.60 | [-17.72, -7.50] | -0.30 | -4.85 | < .001 |
| *RM others* | 1.72 | 1.82 | [-1,86, 5.31] | 0.06 | 0.95 | .345 |
| Trust (0-100) in round 1 | 0.10 | 0.04 | [0.03, 0.17] | 0.15 | 2.44 | .008 |
| Defection by the responder (1, 0) in round 1 | -9.66 | 1.82 | [-13.28, -6.08] | -0.23 | -4.96 | < .001 |
| Amount paid to punish the responder (0-50) in round 1 | 0.13 | 0.07 | [-0.01, 0.27] | 0.09 | 1.55 | .072 |
| Condition x Society | 6.30 | 3.44 | [-0.47, 13.06] | 0.13 | 1.83 | .068 |
| Society x *RM others* | -6.23 | 2.69 | [-11.51, -0.94] | -0.13 | -2.32 | .021 |
| Trust in R1 (round 1) x Defection by the responder in R1 | 0.12 | 0.06 | [0.01, 0.23] | 0.13 | 2.17 | .030 |
| Trust in R1 x Amount paid to punish the responder in R1 | -0.01 | 0.00 | [-0.01, -0.002] | -0.13 | -2.81 | .005 |
| Note. Adjusted R^2^ = 0.18. CI = confidence interval for *b*. VIF values were < 3.11. | | | | | | |

There were two interactions involving *Trust* in round 1. First, *Trust* in round 1 interacted with whether the partner defected in round 1 (β = .13, *p* = .030). *Trust* in round 1 increased *Reciprocation by the participant* in round 2, even more when the responder had defected in round 1 than when the responder had reciprocated. The second interaction was between *Trust* in round 1 and *Amount paid to punish the responder* in round (β = -.13, *p* = .005). Those who trusted more and punished more in round 1 reciprocated less in round 2. These two interactions had opposite effects on reciprocation in round 2.

Effects of other predictors are mostly in line with the main analysis for *Reciprocation by the participant* in round 1 (see S3 Appendix). Note that *RM others* (β = .06, *p* = .345) did not significantly increase *Reciprocation by the participant* in round 2, when participants already had direct experiences of their current partner. There was, however, a significant interaction between Society and *RM others* (β = -.13, *p* = .021), suggesting that Americans who thought others had more opportunities to form new relationships reciprocated less. However, this effect of *RM others* did not significantly decrease reciprocation when analyzing American participants alone, controlling for the other variables (β = -.09, *p* = .105).

**S5.2.1 Reciprocation in round 2 in the US and Japan**

As we found interactions involving society, we separately analyzed American (*n* = 249) and Japanese (*n* = 260) participants. We again found that the behaviors in round 1 affected *Reciprocation by the participant* in round 2. Both American and Japanese participants reciprocated less in round 2 when the partner had defected on them in round 1 than when the partner had reciprocated (US: β = -.26, *p* = 10^-5^, see Table S10-2; Japan: β = -.20, *p* = .0008, see Table S10-3).

| **S10-2 Table. Factors affecting reciprocation by American participants in round 2.** | | | | |  |  | |
| --- | --- | --- | --- | --- | --- | --- | --- |
| Predictors | *b* | *SE* | 95% CI | *β* | *t* | *p* |  |
| *RM others* | -3.41 | 2.08 | [-7.51, 0.70] | -0.10 | -1.63 | .104 |  |
| Trust (0-100) in round 1 | 0.13 | 0.04 | [0.05, 0.20] | 0.20 | 3.28 | .001 |  |
| Defection by the responder (1, 0) in round 1 | -11.36 | 2.66 | [-16.60, -6.11] | -0.26 | -4.27 | < .001 |  |
| Amount paid to punish the responder (0-50) in round 1 | 0.26 | 0.09 | [0.08, 0.44] | 0.19 | 2.79 | .006 |  |
| Trust in R1 x Amount paid to punish the responder in R1 | -0.01 | 0.00 | [-0.01, -0.002] | -0.19 | -2.87 | .005 |  |
| Note. Adjusted R^2^ = 0.16. CI = confidence interval for *b*. VIF values were < 1.39. | | | | | | | |

| **S10-3 Table. Factors affecting reciprocation by Japanese participants in round 2.** | | | | |  | |  | |  |
| --- | --- | --- | --- | --- | --- | --- | --- | --- | --- |
| Predictors | *b* | *SE* | 95% CI | *β* | | *t* | | *p* | |
| Condition: High Partner Choice (vs. Low) | -3.28 | 2.31 | [-7.53, 1.28] | -0.08 | | -1.42 | | .158 | |
| Trust (0-100) in round 1 | 0.08 | 0.05 | [-0.02, 0.17] | 0.12 | | 1.58 | | .115 | |
| Defection by the responder (1, 0) in round 1 | -6.05 | 2.38 | [-12.38, -3.28] | -0.20 | | -3.39 | | .001 | |
| Trust in R1 x Defection by the responder in R1 | 0.22 | 0.07 | [0.08, 0.36] | 0.23 | | 3.03 | | .003 | |
| Note. Adjusted R^2^ = 0.15. CI = confidence interval for *b*. VIF values were < 1.83. | | | | | | | | |  |

There were some effects that were found only in one of the societies. In the US (see S10-2 Table), those who had sent more points to the partner in round 1 reciprocated more in round 2 (*Trust*: β = .20, *p* = .001). Also, those who paid more points to punish the partner in round 1 returned more points in round 2 (*Amount paid to punish the responder*: β = .19, *p* = .006). However, these main effects were qualified by an interaction between *Trust* and *Amount paid to punish the responder* (β = -.19, *p* = .005). Those who trusted more and then paid more to punish the partner in round 1 reciprocated less in round 2.

The interaction between society and *RM others* (β = -.13, *p* = .021; see S10-1 Table) suggests that *RM* *others* might decrease R*eciprocation by the participant* only in Americans. However, when analyzing only American participants, *RM others* did not significantly decrease *Reciprocation* *by the participant* in round 2, controlling for the other variables (β = -.10, *p* = .104).

In Japan (see S10-3 Table), there was no significant effect of *Trust* in round 1 on *Reciprocation by the participant* in round 2 (β = .12, *p* = .115). However, there was a significant interaction between *Trust* and *Defection by the responder* in round 1 (β = .23, *p* = .003). Those who had trusted more in round 1 reciprocated even more when the responder had defected in round 1 than when the responder had reciprocated.

Overall, across two societies, those who experienced reciprocation in round 1 reciprocated in round 2. We did not find any significant effect of *RM others* or condition on reciprocation in round 2. Once participants had experienced how their partner treated them in round 1, their motivations to reciprocate were mostly regulated by these prior experiences.

**S5.3 Trust in round 2**

Lastly, we examine what predicts *Trust* in round 2. We test whether those who had been punished by the partner in round 1 sent fewer points to their punitive partners in round 2.

The DV was *Trust* in round 2: how many points the participant sent to their partner (0-100). Because we were interested in the effect of having been punished in round 1, this analysis is restricted to those who were at risk of punishment (i.e., those who returned 40% or less in round 1; *n* = 236).

We controlled for behaviors in round 1: how many points participants reciprocated (*Reciprocation by the participant* in round 1: 0-40%) and whether the partner (truster) punished the participant (*Punishment received* in round 1: 1 = punished, 0 = not punished). We also controlled for condition (High Partner Choice = 1, Low Partner Choice = 0), society (US = 1, Japan = 0), and *RM others*.

Table S11-1 presents the effects of predictors on *Trust* in round 2 for those who could have been punished in round 1. Being punished by the partner in round 1 significantly decreased the points participants sent to their partner in round 2 (*Punishment received*: β = -.22, *p* = .0002). We also found that American participants tended to risk more points than Japanese participants (Society: β = .22, *p* = .005), which corroborates existing theories about the function of trust in different social ecologies [71].

| **S11-1 Table. Factors affecting trust in round 2 for those who could have been punished in round 1 (for returning 40% or less).** | | | | | | |
| --- | --- | --- | --- | --- | --- | --- |
| Predictors | *b* | *SE* | 95% CI | *β* | *t* | *p* |
| Condition: High Partner Choice (vs. Low) | 10.64 | 5.96 | [-1.11, 22.39] | 0.16 | 1.79 | .076 |
| Society: US (vs. Japan) | 14.96 | 5.24 | [4.63, 25.28] | 0.22 | 2.86 | .005 |
| Reciprocation by the participant (0-40) in round 1 | 1.40 | 0.20 | [0.99, 1.80] | 0.55 | 6.86 | < .001 |
| Punishment received (0, 1) in round 1 | -14.69 | 3.86 | [-22.30, -7.09] | -0.22 | -3.81 | < .001 |
| Condition x Society | -14.53 | 7.89 | [-30.08, 1.02] | -0.19 | -1.84 | .067 |
| Reciprocation in R1 x Punishment received in R1 | -0.83 | 0.30 | [-1.41, -0.25] | -0.22 | -2.80 | .006 |
| Note. Adjusted R^2^ = 0.22. CI = confidence interval for *b*. VIF values were < 3.32. | | | | | | |

Those who had reciprocated in round 1 sent more points to the partner in round 2 (*Reciprocation by the participant*: β = .55, *p* = 10^-11^; see Table S11-1). However, there was a significant interaction between *Reciprocation by the participant* in round 1 and *Punishment received* in round 1 (β = -.22, *p* = .006): Those who had reciprocated more became less trusting when the partner had punished them.

Because those who returned 40% had provided a positive payoff to their partner, they could have felt wronged by being punished (see the main text Section 3.1.1). So, we conducted separate analyses for those who reciprocated by returning 40% (*n* = 83) and those who defected by returning less than 40% (*n* = 153).

For those who reciprocated by returning 40% in round 1, being punished by the partner significantly decreased their *Trust* in round 2 (*Punishment received*: β = -.41, *p* = .0001; see Table S11-2). This was the only significant predictor that remained in the model.

On the other hand, for those who defected by giving their partner a negative payoff (by returning 0-30%), being punished did not significantly decrease their *Trust* in round 2 (*Punishment received*: β = -.12, *p* = .099; see Table S11-3). In this subsample, we again found that those who had reciprocated more in round 1 risked more points in round 2 (*Trust*: β = .42, *p* = 10^-8^) and American participants risked more points than Japanese participants (Society: β = .22, *p* = .022).

| **S11-2 Table. Factors affecting trust in round 2 for those who returned 40% in round 1.** | | | | | | |
| --- | --- | --- | --- | --- | --- | --- |
| Predictors | *b* | *SE* | 95% CI | *β* | *t* | *p* |
| Condition: High Partner Choice (vs. Low) | 11.45 | 6.55 | [-1.59, 24.50] | 0.17 | 1.75 | .084 |
| Society: US (vs. Japan) | 12.21 | 6.95 | [-1.64, 26.06] | 0.19 | 1.76 | .083 |
| *RM others* | -9.71 | 7.18 | [-24.01, 4.59] | -0.20 | -1.35 | .180 |
| Punishment received (0, 1) in round 1 | -26.87 | 6.56 | [-39.93, -13.80] | -0.41 | -4.10 | < .001 |
| Society x *RM others* | 14.64 | 10.60 | [-6.46, 35.74] | 0.19 | 1.38 | .171 |
| Note. Adjusted R^2^ = 0.19. CI = confidence interval for *b*. VIF values were < 2.12. | | | | | | |

| **S11-3 Table. Factors affecting trust in round 2 for those who returned 30% or less in round 1.** | | | | | | |
| --- | --- | --- | --- | --- | --- | --- |
| Predictors | *b* | *SE* | 95% CI | *β* | *t* | *p* |
| Condition: High Partner Choice (vs. Low) | 11.49 | 7.67 | [-3.67, 26.66] | 0.18 | 1.50 | .136 |
| Society: US (vs. Japan) | 14.70 | 6.35 | [2.15, 27.25] | 0.22 | 2.32 | .022 |
| Reciprocation by the participant (0-30) in round 1 | 1.21 | 0.21 | [0.80, 1.62] | 0.42 | 5.80 | < .001 |
| Punishment received (0, 1) in round 1 | -7.95 | 4.79 | [-17.41, 1.50] | -0.12 | -1.66 | .099 |
| Condition x Society | -22.55 | 9.87 | [-42.05, -3.05] | -0.31 | -2.29 | .024 |
| Note. Adjusted R^2^ = 0.20. CI = confidence interval for *b*. VIF values were < 3.58. | | | | | | |

Overall, these results validate the pattern found for retaliatory punishment in round 2. In the current study, punishing the partner in round 1 did not elicit better treatment subsequently. Rather, punishment tended to beget retaliation—punishers received more punishment and less trust.

**S5.3.1 Trust in round 2 in the US and Japan**

Because we found interactions involving society, we separately analyzed American (*n* = 133) and Japanese (*n* = 103) participants. Note that we again limited analyses to those who could have been punished.

*Reciprocation by the participant* in round 1 predicted *Trust* in round 2 in both societies: Those who reciprocated more in round 1 sent more points to the partner in round 2 (US: β = .57, *p* = 10^-8^, see Table S11-4; Japan: β = .34, *p* = .0003, see Table S11-5). This was the only significant predictor that remained in the model for Japanese participants (Table S11-5). In American participants, *Punishment received* in round 1 decreased *Trust* in round 2 (β = -.28, *p* = .0003). Also, there was a significant interaction between *Reciprocation by the participant* in round 1 and *Punishment received* in round 1 (β = -.21, *p* = .030): the same effect found when the two societies were analyzed together (see Table S11-1). Those who had reciprocated more became less trusting when the partner had punished them.

| **S11-4 Table. Factors affecting trust in round 2 for Americans who could have been punished in round 1 (for returning 40% or less).** | | | | | | |
| --- | --- | --- | --- | --- | --- | --- |
| Predictors | *b* | *SE* | 95% CI | *β* | *t* | *p* |
| Reciprocation by the participant (0-40) in round 1 | 1.58 | 0.26 | [1.07, 2.09] | 0.57 | 6.09 | < .001 |
| Punishment received (0, 1) in round 1 | -20.91 | 5.63 | [-32.04, -9.77] | -0.28 | -3.72 | < .001 |
| Reciprocation in R1 x Punishment received in R1 | -0.96 | 0.44 | [-1.83, -0.10] | -0.21 | -2.20 | .030 |
| Note. Adjusted R^2^ = 0.26. CI = confidence interval for *b.* VIF values were < 1.59. | | | | | | |

| **S11-5 Table. Factors affecting trust in round 2 for Japanese participants who could have been punished in round 1 (for returning 40% or less).** | | | | | | |  |
| --- | --- | --- | --- | --- | --- | --- | --- |
| Predictors | *b* | *SE* | 95% CI | *β* | *t* | *p* | |
| Condition: High Partner Choice (vs. Low) | 9.85 | 5.20 | [-0.47, 20.17] | 0.17 | 1.89 | .061 | |
| Reciprocation by the participant (0-40) in round 1 | 0.75 | 0.20 | [0.36, 1.15] | 0.34 | 3.76 | < .001 | |
| Punishment received (0, 1) in round 1 | -8.09 | 5.13 | [-18.27, 2.10] | -0.14 | -1.58 | 0.12 | |
| Note. Adjusted R^2^ = 0.16. CI = confidence interval for *b.* VIF values were < 1.02. | | | | | | |  |

**S5.3.2 Trust in round 1**

In the main text we focused on motivations to reciprocate and to punish, so we did not report *Trust* in round 1. Here, we examine what predicts how many points the participants sent to the partner in round 1 (*Trust*: 0-100) (*n* = 509).

Nothing significantly predicted *Trust* in round 1 (see Table S11-6). There were two marginal effects that remained in the model. American participants tended to risk more points than Japanese participants, and *RM others* tended to increase trust.

| **S11-6 Table. Factors affecting trust in round 1.** | | | | | | | |
| --- | --- | --- | --- | --- | --- | --- | --- |
| Predictors | *b* | *SE* | 95% CI | *β* | *t* | *p* |  |
| Society: US (vs. Japan) | 5.99 | 3.17 | [-0.24, 12.23] | 0.09 | 1.89 | .059 |  |
| *RM others* | 4.37 | 2.26 | [-0.07, 8.81] | 0.09 | 1.94 | .054 |  |
| Note. Adjusted R^2^ = 0.02. CI = confidence interval for *b.* VIF values were < 1.02. | | | | | | | |
